# The Construction of lncRNA/circRNA–miRNA–mRNA Networks Reveals Functional Genes Related to Growth Traits in *Schima superba*

**DOI:** 10.3390/ijms25042171

**Published:** 2024-02-11

**Authors:** Qingsong Bai, Lingling Shi, Kejian Li, Fang Xu, Weihua Zhang

**Affiliations:** Guangdong Provincial Key Laboratory of Silviculture, Protection and Utilization, Guangdong Academy of Forestry, Guangzhou 510520, China

**Keywords:** *Schima superba*, non-coding RNA, regulatory network, growth trait, candidate gene

## Abstract

*Schima superba* is a precious timber and fire-resistant tree species widely distributed in southern China. Currently, there is little knowledge related to its growth traits, especially with respect to molecular breeding. The lack of relevant information has delayed the development of modern breeding. The purpose is to identify probable functional genes involved in *S. superba* growth through whole transcriptome sequencing. In this study, a total of 32,711 mRNAs, 525 miRNAs, 54,312 lncRNAs, and 1522 circRNAs were identified from 10 *S. superba* individuals containing different volumes of wood. Four possible regulators, comprising three lncRNAs, one circRNA, and eleven key miRNAs, were identified from the regulatory networks of lncRNA–miRNA–mRNA and circRNA–miRNA–mRNA to supply information on ncRNAs. Several candidate genes involved in phenylpropane and cellulose biosynthesis pathways, including *Ss4CL2*, *SsCSL1*, and *SsCSL2*, and transcription factors, including *SsDELLA2* (*SsSLR*), *SsDELLA3* (*SsSLN*), *SsDELLA5* (*SsGAI-like2*), and *SsNAM1*, were identified to reveal the molecular regulatory mechanisms regulating the growth traits of *S. superba*. The results not merely provide candidate functional genes related to *S. superba* growth trait and will be useful to carry out molecular breeding, but the strategy and method also provide scientists with an effective approach to revealing mechanisms behind important economic traits in other species.

## 1. Introduction

*Schima superba* is an important native broad-leaved tree species widely distributed in southern China that provides people with timber for building materials and ecological protection from forest fire [1,2]. Continuous scientific studies have concentrated on this species over the past two decades and include genetic map construction [2], population structure and genetic diversity analysis of germplasm resources [1], and conventional breeding work [1,2]. However, a lack of knowledge of the molecular mechanism of specific trait formation has hindered the development of molecular genetic improvements and applications.

Forest tree growth traits are usually controlled by specific functional and regulatory genes and related pathways [3,4,5,6,7]. Among the numerous regulatory mechanisms and pathways, long non-coding RNA (lncRNA) and circular RNA (circRNA) are core factors that regulate specific traits through microRNAs (miRNAs) [8]. LncRNAs and circRNAs usually play roles through miRNAs as mediators to control the expression of mRNAs. Two types of regulation models, namely lncRNA–miRNA–mRNA and circRNA–miRNA–mRNA, are usually involved in specific traits and physiological processes [6,9]. miRNAs can discriminate and degrade target genes through complementary mechanisms to directly regulate plant traits [10,11,12].

This type of regulatory mechanism has been discovered in many plant and forest species, such as the identification of lncRNA–miRNA–mRNA regulatory modules of cold stress in *Ammopiptanthus nanus* [13], the development of embryos via a lncRNA–miRNA–mRNA network in *Picea glauca* [14], the regulation of floral development in *Glycine max* via a circRNA–miRNA–mRNA network [15], and the contribution of a circRNA–miRNA–mRNA network to cold tolerance in *Camellia sinensis* [16]. As the most important genetic and improvement targets, growth traits have long been the focus of forest researchers to produce more forest products and increase economic benefits. Mature sequencing technology has increased the possibility of capturing key information on specific traits and physiological processes. In recent years, whole transcriptome sequencing has provided scientists with enough information to reveal specific traits of tree species.

In this study, whole transcriptome sequencing was employed to carry out an in-depth analysis of the growth traits in *S. superba*. Ten individuals with different volumes of wood (VW) were selected and used for whole transcriptome sequencing. Differentially expressed lncRNAs, circRNAs, miRNAs, and mRNAs identified from the whole transcriptome data will be used to construct RNA regulatory networks (lncRNA–miRNA–mRNA and circRNA–miRNA–mRNA) relevant to growth traits. It is expected to select core lncRNAs, circRNAs, and miRNAs that serve as the key factors controlling the growth traits of *S. superba*. Then, candidate genes will be identified and used for qRT-PCR validation to ascertain their crucial roles in this process. Finally, due to the different genetic backgrounds, DNA allelic variations associated with growth traits will also be identified and may be used for marker-assisted selection. Based on these analyses, researchers can better understand the molecular mechanism of the tremendous differences in growth traits to carry out molecular breeding work in the future.

## 2. Results

### 2.1. Quality and Statistics of Whole Transcriptome Sequencing

In this study, 10 *S. superba* individuals with different VW values were used for whole transcriptome sequencing, including mRNA, miRNA, lncRNA, and circRNA. After RNA sequencing, an average number of 89,420,740 raw reads and 88,513,958 clean reads were obtained for the identification of lncRNAs, mRNAs, and circRNAs. And an average number of 13.28 Gb clean data were finally obtained (Table S1). An average number of 12,455,427 raw reads and 12,243,183 clean reads were obtained for the identification of miRNAs. And an average number of 0.62 Gb clean data were finally obtained (Table S2).

**Figure 1:**
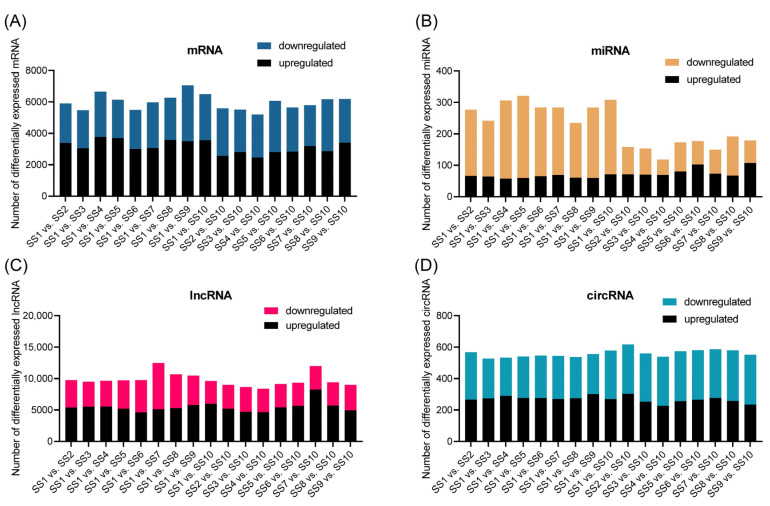
Statistics of differentially expressed mRNAs (DEmRNAs), miRNAs (DEmiRNAs), lncRNAs (DElncRNAs), and circRNAs (DEcircRNAs). (**A**–**D**) Differential analysis was conducted between SS1 and SS10 with the other samples. Column diagrams represent the number of DEmRNAs, DEmiRNAs, DElncRNAs, and DEcircRNAs using SS1 and SS10 as reference samples, respectively.

Finally, a total of 32,711 mRNAs, 525 miRNAs, 54,312 lncRNAs, and 1522 circRNAs were identified from the 10 samples (Figure S1). Overall, similar distribution trends were detected among the ten samples for the four types of RNAs. Comparative analyses of these four types of RNA were conducted between any pair of samples. The results of comparative analyses that set SS1 (with the highest VW) and SS10 (with the lowest VW) are displayed in Figure 1. The comparative analysis results of 17 pairs showed that there were at least 2455 upregulated and 2433 downregulated mRNAs in the pairs SS4 vs. SS10 and SS1 vs. SS3, respectively (Figure 1A). Based on miRNA-seq analysis, there were at least 57 upregulated and 49 downregulated miRNAs in the pairs SS1 vs. SS4 and SS4 vs. SS10, respectively (Figure 1B). In addition, at least 4631 upregulated and 3651 downregulated lncRNAs were discovered in pairs SS1 vs. SS6 and SS1 vs. SS10, respectively (Figure 1C). A total of at least 226 upregulated and 243 downregulated circRNAs were identified in the pairs SS4 vs. SS10 and SS1 vs. SS4 (Figure 1D). GO classifications of DEmRNAs and targets of DElncRNAs, DEmiRNAs, and DEcircRNAs revealed similar results between comparisons of using SS1 and SS10 as reference samples, respectively (Figures S2–S5). The GO terms of DEmRNAs are mainly concentrated on molecular functions, while the GO terms of the targets of DElncRNAs, DEmiRNAs, and DEcircRNAs are concentrated on biological processes. The KEGG pathway enrichment of the DEmRNAs was mainly focused on phenylpropanoid biosynthesis, starch and sucrose metabolism, carbon metabolism, and plant hormone signal transduction (Figure S6A). The targets of DEmiRNAs, DElncRNAs, and DEcircRNAs were mainly focused on the biosynthesis of secondary metabolites, carbon metabolism, plant hormone signal transduction, and metabolic pathways (Figure S6B–D).

### 2.2. Regulatory Networks of lncRNA-miRNA-mRNA

Comparative analyses were conducted between any pair of samples to identify DElncRNAs, DEmiRNAs, and DEmRNAs. Only the comparison pairs that were simultaneously identified in at least six groups relative to the reference sample were treated as valid connections. A total of 755 DElncRNAs, 168 DEmiRNAs, and 457 DEmRNAs were identified in comparison to SS1 for a total of 2041 connections, with 1181 upregulated between 698 DElncRNAs and 134 DEmiRNAs, 747 upregulated between 137 DEmiRNAs and 414 DEmRNAs, 66 downregulated between 57 DElncRNAs and 21 DEmiRNAs, and 47 downregulated between 21 DEmiRNAs and 43 DEmRNAs (Figure S7). A total of 19 DEmiRNAs, including aly-miR157d-3p, aly-miR172e-3p, ata-miR395b-3p, ath-miR156a-5p, bdi-miR159a-3p, fve-miR156h, gma-miR172b-5p, gma-miR396a-3p, gma-miR6300, hbr-miR156, novel_102, osa-miR396a-3p, osa-miR5083, pab-miR156b, pta-miR319, rgl-miR5139, sbi-miR172b, stu-miR156f-5p, and vca-miR535-3p, were regarded as key miRNAs due to their connections with the greatest number of DElncRNAs and DEmRNAs (Table S3). The relative expression of TPM values across the 10 individuals is shown in Figure S8. The expression of these miRNAs in SS1 was lower than in the other individuals.

**Figure 2:**
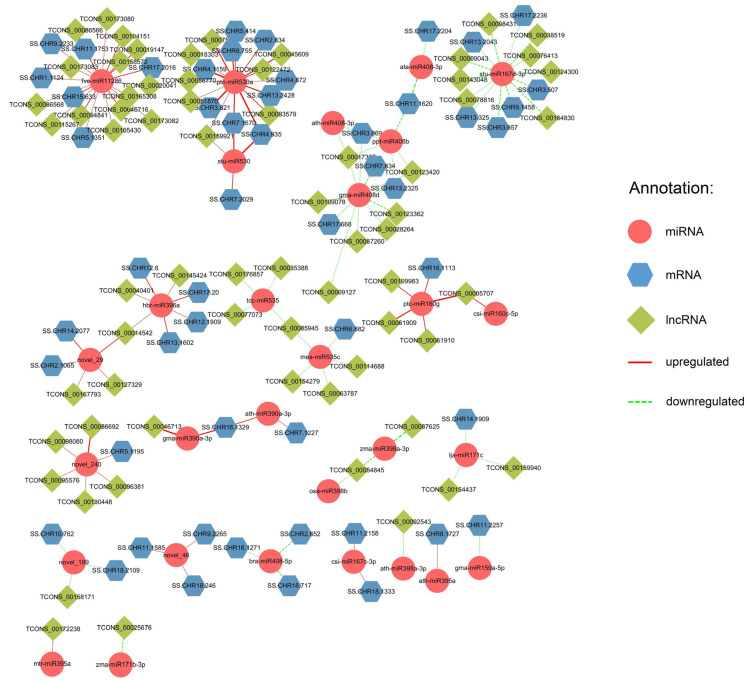
Regulatory network of lncRNA–miRNA–mRNA compared to SS10. Differential analysis was conducted between the individual with the lowest volume of wood (SS10) and other nine samples by using SS10 as reference sample. Across nine comparison pairs, the three types of differential RNAs that were detected in at least six pairs were used for construction of the regulatory network. Solid red lines represent upregulated RNAs, while dotted green lines represent downregulated RNAs. The thicker the line, the higher the number of comparison pairs.

In addition, 67 DElncRNAs, 29 DEmiRNAs, and 52 DEmRNAs were identified in comparison to SS10, for a total of 134 connections, with 41 upregulated between 38 DElncRNAs and 10 DEmiRNAs, 35 upregulated between 12 DEmiRNAs, and 32 DEmRNAs, 34 downregulated between 29 DElncRNAs and 12 DEmiRNAs and 24 downregulated between 10 DEmiRNAs and 20 DEmRNAs (Figure 2). Only four DEmiRNAs, fve-miR11286, gma-miR408d, ptc-miR530a, and stu-miR167d-3p, with the largest number of DElncRNAs and DEmRNAs, are shown in Figure 2 and Table S4.

### 2.3. Regulatory Networks of circRNA–miRNA–mRNA

**Figure 3:**
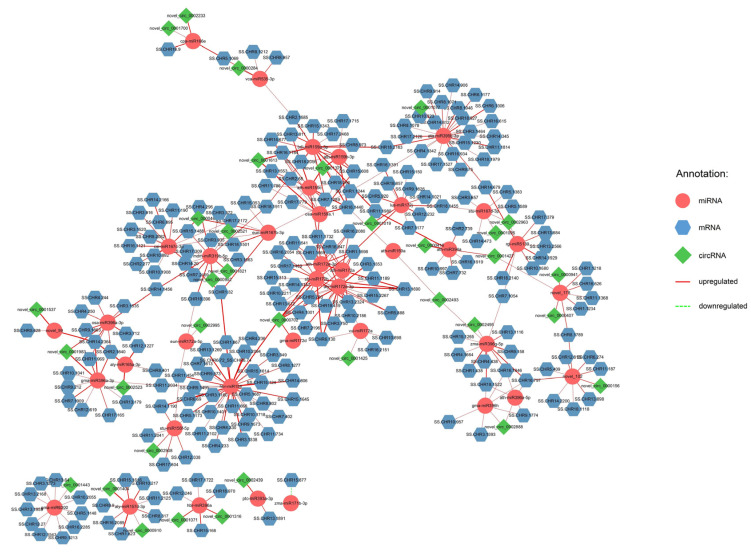
Regulatory network of circRNA–miRNA–mRNA compared to SS1. Differential analysis was conducted between the individual with the highest volume of wood (SS1) and other nine samples using SS1 as the reference sample. Across nine comparison pairs, the three types of differential RNAs that were detected in at least six pairs were used for the construction of the regulatory network. Solid red lines represent upregulated RNAs, while dotted green lines represent downregulated RNAs. The thicker the line, the higher the number of comparison pairs.

Similar to the construction of the lncRNA–miRNA–mRNA network, comparative analysis was also conducted on any two samples to identify DEcircRNAs, DEmiRNAs, and DEmRNAs. A total of 34 DEcircRNAs, 38 DEmiRNAs, and 221 DEmRNAs were identified in comparison to SS1 for a total of 349 connections, including 47 upregulated between 34 DEcircRNAs and 37 DEmiRNAs and 301 upregulated between 36 DEmiRNAs and 220 DEmRNAs. Only one downregulated connection was identified between DEmiRNA and DEmRNA (Figure 3). A total of 16 DEmiRNAs, including aly-miR157d-3p, aly-miR172e-3p, ata-miR395b-3p, ath-miR159c, bdi-miR159a-3p, csi-miR167c-3p, gma-miR396a-3p, gma-miR6300, hbr-miR156, lus-miR172j, mdm-miR319b-5p, novel_102, osa-miR159a.1, osa-miR396a-3p, sbi-miR172b, and zma-miR396g-5p, were regarded as key miRNAs due to their connections with the largest number of DEcircRNAs and DEmRNAs (Table S5). The relative expression of TPM values across the 10 individuals is shown in Figure S9. Similar to the lncRNA–miRNA–mRNA results, the expression of these miRNAs in SS1 was also lower than in the other individuals.

**Figure 4:**
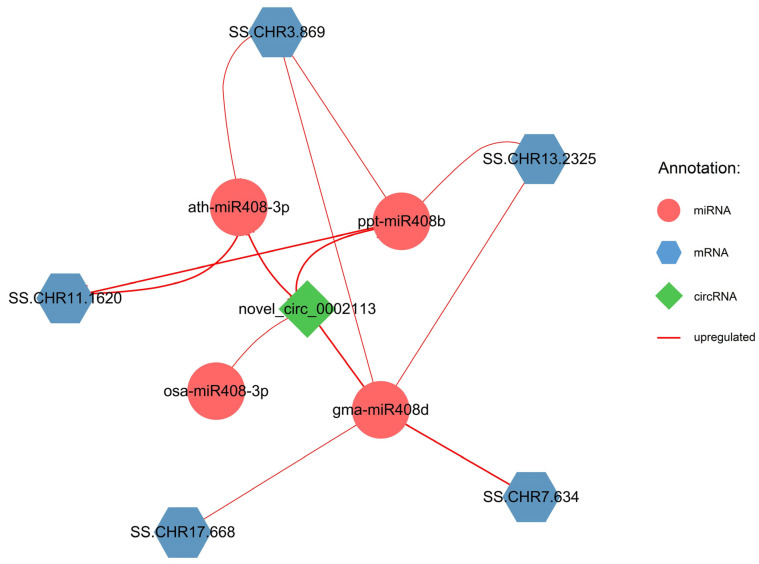
Regulatory network of lncRNA–miRNA–mRNA compared to SS10. Differential analysis was conducted between the individual with the lowest volume of wood (SS10) and other nine samples using SS10 as the reference sample. Across nine comparison pairs, the three types of differential RNAs that were detected in at least six pairs were used for the construction of the regulatory network. Solid red lines represent upregulated RNAs. The thicker the line, the higher the number of comparison pairs.

Only downregulated connections were identified in comparison to SS10. A total of one DEcircRNA, four DEmiRNAs, and five DEmRNAs were identified from these comparison pairs. Four upregulated connections were identified between one DEcircRNA and four DEmiRNAs, and nine upregulated connections were identified between three DEmiRNAs and five DEmRNAs (Figure 4). Only three DEmiRNAs, ath-miR408-3p, gma-miR408d, and ppt-miR408b, with the largest number of DElncRNAs and DEmRNAs, are shown in Figure 4 and Table S6.

After statistical analysis, 10 DEmiRNAs, namely aly-miR157d-3p, aly-miR172e-3p, ata-miR395b-3p, bdi-miR159a-3p, gma-miR396a-3p, gma-miR6300, hbr-miR156, novel_102, osa-miR396a-3p, and sbi-miR172b, were identified and regarded as key miRNAs in the regulatory networks of lncRNA–miRNA–mRNA and circRNA–miRNA–mRNA in comparison to SS1. Only one DEmiRNA, gma-miR408d, was identified and regarded as a key miRNA in comparison to SS10.

The predicted secondary structures of key miRNAs in comparison to SS1 are displayed in Figure S10. In addition, a correlation analysis was conducted directly among lncRNAs or circRNAs, miRNAs, and mRNAs. Only the lncRNAs showed significant correlations (*p* < 0.01) with miRNAs. The results of correlation coefficients greater than 0.8 or less than −0.8 are displayed in Figure S11. A total of 120 connections were constructed between lncRNAs or mRNAs and miRNAs.

The phenotype data HT, DBH, and VW were used for the correlation analysis with RNA expression in branches and leaves, respectively. Across all related lncRNAs and circRNAs in this analysis, trait VW accounted for the most significantly correlated lncRNAs and circRNAs in the group of comparisons with SS1 (Figure S12). A total of 170 lncRNAs and 96 circRNAs were correlated with VW. Trait DBH accounted for the lowest number of correlations, with 21 lncRNAs and 12 circRNAs, in comparison to SS1.

**Figure 5:**
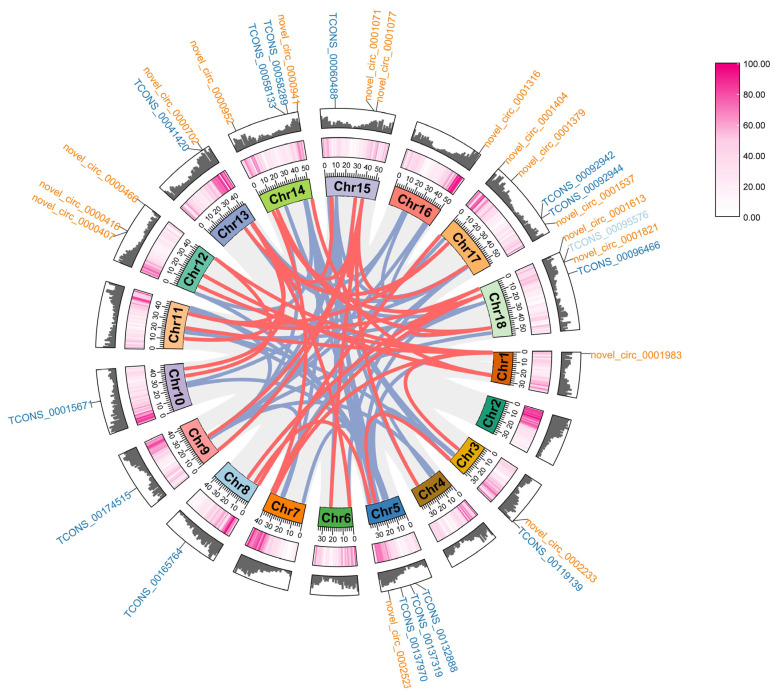
Chromosome distributions and connections of key lncRNAs, circRNAs, and mRNAs. The lncRNAs, circRNAs, and mRNAs were aligned to an assembled chromosome-scale *Schima superba* reference genome. Gene density was displayed using heatmaps and columns. The darker the color in the heatmap or the higher the column, the higher the gene density. Key lncRNAs and circRNAs with FPKM and TPM > 5 are displayed on the chromosomes. Gene IDs in blue represent lncRNAs. Gene IDs in yellow represent circRNAs. Connections between lncRNAs and mRNAs are displayed using blue curves. Connections between circRNAs and mRNAs are displayed using red curves. Connections between lncRNAs or circRNAs and other mRNAs are displayed using grey curves.

A circos plot was used to show the relationship between lncRNAs or circRNAs and mRNAs (Figure 5). A total of 12,657 and 342 connections were identified, from 749 lncRNAs and 35 circRNAs to 475 and 191 mRNAs, respectively. All lncRNAs and mRNAs belonging to lncRNA–miRNA–mRNA networks and mRNAs belonging to circRNA–miRNA–mRNA networks were distributed widely on all 18 chromosomes across the *S. superba* genome, while circRNAs were distributed only on 15 chromosomes. Among these connections, 15 main lncRNAs (TCONS_00015671, TCONS_00137319, TCONS_00060488, TCONS_00095576, TCONS_00119139, TCONS_00058133, TCONS_00096466, TCONS_00165764, TCONS_00041420, TCONS_00132888, TCONS_00092944, TCONS_00092942, TCONS_00137970, TCONS_00174515, and TCONS_00058289) distributed on 10 chromosomes, including one lncRNA (TCONS_00095576) identified from comparisons to both SS1 and SS10, and 17 main circRNAs (novel_circ_0001821, novel_circ_0001071, novel_circ_0000466, novel_circ_0002523, novel_circ_0001379, novel_circ_0000702, novel_circ_0002233, novel_circ_0000416, novel_circ_0000952, novel_circ_0001537, novel_circ_0001613, novel_circ_0000407, novel_circ_0000941, novel_circ_0001077, novel_circ_0001316, and novel_circ_0001404, novel_circ_0001983) distributed on 10 chromosomes were connected with mRNAs using correlation analysis. The expressions of these 15 DElncRNAs and 17 DEcircRNAs in SS1 were notably higher than in the other nine samples (Figures S13 and S14). In total, 64 and 37 potential genes from mRNAs, including the transcription factors *WOX*, *TCP*, *MYB*, *Dof*, *NAC*, *F-box*, *LOB*, *DELLA*, and *AP2/ERF,* and the functional genes, namely flavonoid 3’,5’-hydroxylase, cytochrome P450, and cellulose synthase, were identified.

### 2.4. Identification and Characteristics of Candidate Genes

**Figure 6:**
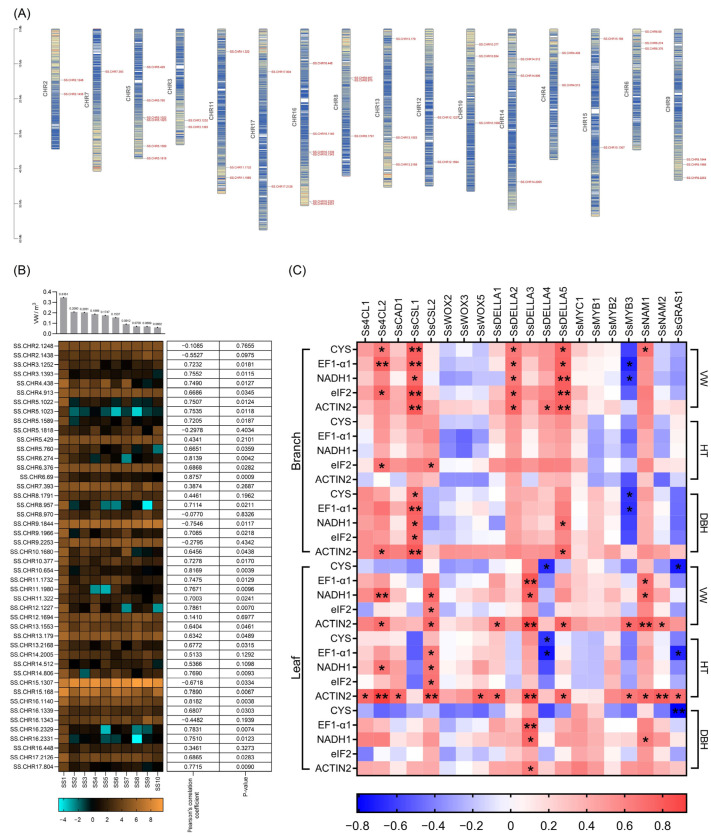
Identification and expression of candidate genes. (**A**) Chromosome distribution of candidate genes. (**B**) Relationship between gene expression and growth traits. A heatmap was used to show the expression levels of each candidate gene (FPKM ≥ 5) across the 10 individuals. Phenotype values of the volume of wood for the 10 individuals are displayed above the heatmap. Correlation coefficients and *p*-value of each candidate gene are displayed on the right side. (**C**) qRT-PCR validation and correlation analysis of 20 candidate genes associated with growth traits, including volume of wood (VW), height (HT), and diameter at breast height (DBH), using five reference genes, CYS, EF1-α1, NADH1, eIF2, and ACTIN2. Significance correlation: * represents *p* < 0.05; ** represents *p* < 0.01. Red represents positive correlation between phenotype and expression; blue represents negative correlation.

In addition to the above correlation analysis among the lncRNA–miRNA–mRNA and circRNA–miRNA–mRNA networks, correlation analysis between RNA expression and phenotype values was also used to identify candidate genes. A total of 46 candidate genes were identified from the lncRNA–miRNA–mRNA and circRNA–miRNA–mRNA networks (Figure 6). These genes were distributed on 16 of 18 chromosomes across the *S. superba* genome (Figure 6A). The two-tailed correlation analysis indicated that, among these candidate genes, 36 genes were positively associated with the trait VW with a correlation coefficient greater than 0.6 (*p* < 0.05), and three genes were negatively associated with the trait VW with a correlation coefficient greater than 0.6 (*p* < 0.05) (Figure 6B). A total of twenty candidate genes were selected for qRT-PCR validation using five reference genes, among which the expression levels of seven candidate genes (*Ss4CL2*, *SsCSL1*, *SsCSL2*, *SsDELLA2* (*SsSLR*), *SsDELLA3* (*SsSLN*), *SsDELLA5* (*SsGAI-like2*), and *SsNAM1*) presented gradual downward trends from the branches or leaves of individuals with high to low VWs, HTs, and DBH and were significantly positively correlated (*p* < 0.05) with phenotype values of these traits using the five reference genes (Figure 6C, Figures S15 and S16).

### 2.5. Identification of Allelic Variations

The RNA-seq data were used for the identification of SNPs across the 10 samples. After quality control and SNP filtering with a minor allele frequency (MAF) ≥ 0.1, 86,922 SNPs were obtained from the transcriptome data. Information on SNP types and the frequency of each type is described in Table 1. The frequency of transition SNPs (53,283 SNPs; 61.30%) was greater than (nearly 1.58 times) that of transversion SNPs (33,639 SNPs; 38.70%). Transitions of C/T were the most common SNP type (30.72%), while transversions of G/C were the least common SNP type (8.72%). Transitions of C/T were approximately equal to those of A/G, and transversion SNPs did not show excessive proportion variations (ranging from 8.72 to 10.54%).

### 2.6. Phylogenetic Analysis of Cellulose Synthase and Cellulose Synthase-like Proteins

**Figure 7:**
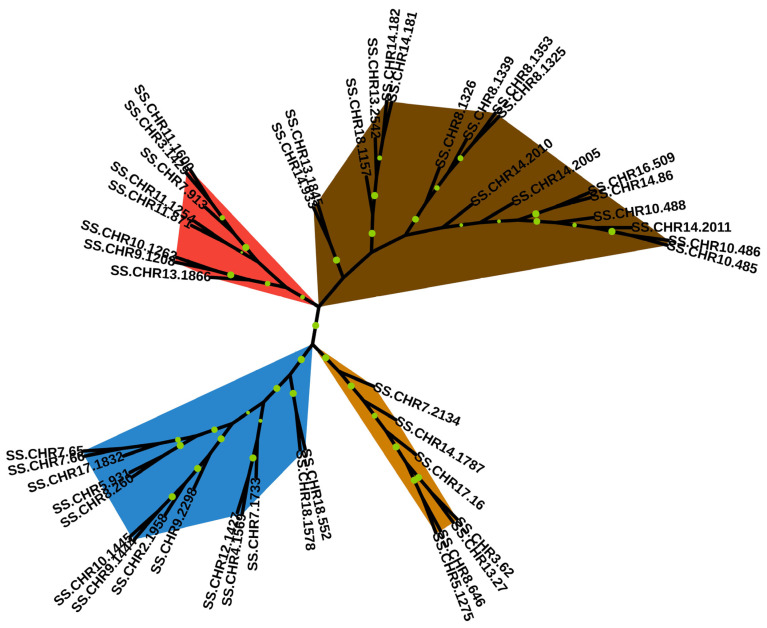
Phylogenetic relationship among cellulose synthase and cellulose synthase-like proteins. A total of 38 related proteins were identified and the full-length proteins were used to construct the phylogenetic tree. The maximum likelihood method in Mega X with 1000 bootstraps was used to conduct the analysis. Colors indicated four main clusters. Bootstrap support rates over 60% are displayed on the branches. The larger the circle size, the higher the support rate.

Conserved motif searching and confirmation were used to determine the final family members. A total of 21 cellulose synthases (CeSAs) and 26 cellulose synthase-like proteins (CSLs) were identified from the *S. superba* genome and transcriptome. The lengths of the longest CeSA (SS.CHR10.1262) and CSL (SS.CHR14.933) coding sequences were 6411 and 4188 bp, respectively. Then, an unrooted phylogenetic tree was constructed to display the genetic relationships among these special enzymes (Figure 7). These proteins were divided into four clusters, containing 8, 18, 7, and 14 genes, respectively. Ten sister pairs were identified in the phylogenetic tree and were entirely supported with 100% bootstraps. In addition, the heatmap of CeSAs and CSLs using FPKM values, conserved motif identification, and gene structure analysis was conducted, as shown in Figure S17. A total of 23 genes were highly expressed in at least one individual using FPKM values. Twelve motifs were identified among these genes.

### 2.7. Differentially Expressed Genes in the Phenylpropane Pathway

**Figure 8:**
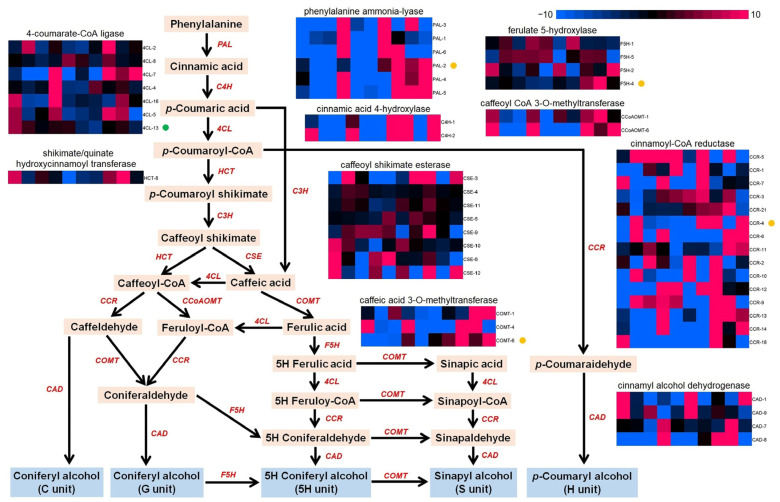
Differentially expressed genes involved in the phenylpropane pathway. PAL, phenylalanine ammonialyase. C4H, cinnamic acid 4-hydroxylase. 4CL, 4-coumarate-CoA ligase. HCT, hydroxycinnamoyl-CoA shikimate/quinate hydroxycinnamoyl transferase. C3’H, p-coumaroyl shikimate 3’hydroxylase. CSE, caffeoyl shikimate esterase. C3H, coumarate 3-hydroxylase. CCR, cinnamoyl-CoA reductase. CCoAOMT, caffeoyl CoA 3-O-methyltransferase. COMT, caffeate/5-hydroxyferulate 3-O-methyltransferase. F5H, ferulate 5-hydroxylase. CAD, cinnamyl alcohol dehydrogenase. Circles on the right side of gene names indicate significant correlations with phenotype values of the trait VW. Green represents positive correlations, while yellow represents negative correlations.

In addition to cellulose biosynthesis, the phenylpropane pathway is another factor that is primarily related to wood properties. Using transcriptomic data, several enzyme genes involved in the phenylpropane pathway were identified, including seven 4-coumarate-CoA ligases (4CLs), one hydroxycinnamoyl-CoA shikimate/quinate hydroxycinnamoyl transferase (HCT), six phenylalanine ammonialyases (PALs), two cinnamic acid 4-hydroxylases (C4Hs), four ferulate 5-hydroxylases (F5Hs), two caffeoyl CoA 3-O-methyltransferases (CCoAOMTs), eight caffeoyl shikimate esterase (CSEs), three caffeate/5-hydroxyferulate 3-O-methyltransferases (COMTs), fifteen cinnamoyl-CoA reductase (CCRs), and four cinnamyl alcohol dehydrogenase (CADs) (Figure 8). Among these DEGs, the FPKM values of 4CL-13 (Ss4CL2) across the 10 samples were positively correlated (correlation coefficient of 0.69) with the phenotype values of VW (*p* < 0.05), while the FPKM values of COMT-6 (correlation coefficient of –0.75) were negatively correlated with VW (*p* < 0.05). In addition, the FPKM values of PAL-2 (–0.69), F5H-4 (–0.71), COMT-6 (–0.76), and CCR-4 (–0.68) were negatively correlated with DBH (*p* < 0.05).

## 3. Discussion

As one of the most important and widely distributed precious broad-leaved tree species in southern China, *S. schima* continuously provides wood material to people due to its dominant wood property of high basic density [17]. The advantage of a stable coefficient of phenotypic variation and a coefficient of genetic variation opens the possibility of increasing the wood yield by regulating growth traits such as HT, DBH, and VW. Therefore, it is urgent to carry out studies related to identifying key genes for regulating growth traits and developing growth trait-associated molecular markers to support molecular breeding.

Non-coding RNAs, such as lncRNAs and circRNAs, have crucial roles in controlling plant growth and development [18,19,20,21]. Whole-transcriptome RNA sequencing can provide scientists with information on pivotal lncRNAs and circRNAs that might be involved in specific physiological processes and potential competing endogenous RNAs (ceRNAs) [22]. Until now, there has been little knowledge of ncRNAs in *S. superba* and other *Camellia* plants [23]. Whole transcriptome information was obtained in this study to identify candidate ncRNAs and molecular networks that are probably involved in *S. superba* growth traits using individuals with different growth phenotypes, which can also provide useful information for the study of *Camellia* plants. In this study, the large number of identified lncRNAs, circRNAs, and miRNAs provides researchers with a database for *S. superba*, among which several lncRNAs, circRNAs, and miRNAs were significantly correlated with growth traits [24,25,26]. In the data analysis procedure, two extreme samples with the highest and lowest values of VW were used as references to conduct comparative analysis. The number of DEmiRNAs in comparison with SS1 was higher than with SS10 and may have been induced by an unbalanced distribution of phenotype values from minimum to maximum. The results also indicated that selecting an individual with the highest value as a reference and carrying out a comparative analysis easily determined candidate regulators in this tree species and directly revealed the molecular networks underlying the target trait. The construction of molecular networks among these types of RNAs and the identification of core candidate RNAs, especially those miRNAs simultaneously identified from lncRNA–miRNA–mRNA and circRNA–miRNA–mRNA networks, were conducive to revealing molecular mechanisms in this species. The lncRNAs TCONS_00049201, TCONS_00121864, and TCONS_00141936 and the circRNA novel_circ_0000702 displayed a higher number of connections with miRNAs than other lncRNAs and circRNAs and should be regarded as potential regulators.

To better understand their functions, regulatory networks were constructed to display ceRNAs among lncRNAs or circRNAs, miRNAs, and mRNAs. Several lncRNAs and circRNAs were selected as core regulators due to their connection numbers with miRNAs and correlation with the phenotype values of VW. miRNAs act like mediators between lncRNAs or circRNAs and mRNAs [6,13,14]. miR157d and miR319 usually modulate the expression of downstream regulators to directly regulate growth and developmental processes in plants [27]. Through regulatory networks, ata-miR395b-3p and gma-miR172b-5p regulated 24 target genes. However, hbr-miR156 regulated 33 target genes, the highest number of genes. Among the core miRNAs, aly-miR157d-3p and pta-miR319 were identified in comparison to SS1, and aly-miR157d-3p was simultaneously identified in the lncRNA–miRNA–mRNA and circRNA–miRNA–mRNA networks. aly-miR172e-3p is also a potential miRNA that might regulate transcription factors implicated in plant growth [28]. There are limited reports of miR395b and miR6300 relevant to plant growth within the scope of existing research [29]. miR159a and miR396a regulate plant growth through their target transcription factors, GRAS, MYB, and GRF [30,31,32]. A similar common phenomenon can be found in miR156 and miR172b [33,34]. Interestingly, the core miRNAs identified in the circRNA–miRNA–mRNA network all belong to the miR408 family. This indicates that this miRNA family, especially gma-miR408d, which was common to both lncRNA–miRNA–mRNA and circRNA–miRNA–mRNA networks, may play important roles in *S. superba* growth [35]. In addition, a novel miRNA, novel_102, was also detected in both the lncRNA and circRNA modules and predicted to have a potential function. Therefore, our study provides useful information on regulatory networks and potential ncRNA regulators related to growth traits in *S. superba*.

ncRNAs are important for regulating plant growth, development, metabolic processes, and stress resistance [6,14,20], and mRNAs that encode active proteins directly participate in the generation of specific traits [36,37]. Transcription factors usually play regulatory roles by modulating the expression of functional genes. In this study, several candidate transcription factors, such as SsDELLA2, SsDELLA3, SsDELLA5, and SsNAM1, were extracted and validated using qRT-PCR. DELLA proteins are core regulators in the plant gibberellin pathway and usually participate in the regulation of growth traits [38,39,40]. In addition, DELLA proteins are involved in the regulation of CeSA gene expression [39]. NAM, which belongs to the NAC transcription factor superfamily, is also usually involved in the regulation of plant growth [41]. Other candidate transcription factors, such as MYB and TCP, were also identified to support the regulatory mechanism. Functional genes, especially those encoding enzyme proteins, are associated with the formation of lignin and cellulose in wood [42]. In this study, genome-wide and transcriptome-wide identification and phylogenetic, gene structure, motif, and expression analyses of cellulose synthesis-related genes and lignin synthesis-related genes were conducted to better clarify the family members participating in wood formation. The differentially expressed genes in the phenylpropane pathway and the cellulose biosynthesis pathway provide new insights into explaining wood formation. Several members, Ss4CL2, SsCSL1, and SsCSL2, are likely relevant to the phenotype values across the 10 individuals, indicating that these enzymes are potential major functional genes in the biosynthesis of cellulose and lignin. Although there is a limited scale of sequencing data, the comparative analyses among 10 individuals still provide us with confidential results related to growth traits in *S. superba*. In the next study, the candidate functional genes can be used for in-depth experimental validation to better reveal the molecular mechanism underlying growth traits.

Allelic variations are important for distinguishing individuals or species. SNPs are one of the latest and most effective allelic variation types that have been widely evaluated in plant species according to population genetic diversity analysis [1]. In addition, the development of target trait-associated allelic variation SNPs identified from the genome and transcriptome is also important work and usually has potential application prospects in tree species breeding [43,44,45,46,47,48]. Except for SNPs distributed across noncoding regions of the genome, SNPs on expressed tags may be closer to related traits. In this work, many SNPs were identified from the transcriptome data and displayed a higher frequency of transition SNPs than transversion SNPs, which was consistent with findings in several other plant species [49,50]. This indicates that allelic variations extracted from expressed transcripts are equal to genomic levels [1]. This also suggests that transitions are better tolerated than transversions in the expansion and diversification processes of this species [51]. Unique allelic variations belonging to samples SS1 or SS10 may play important roles in selecting individuals with superior growth phenotypes. Therefore, continuous work is needed in the future to select trait-associated molecular markers to realize marker-assisted selection. In addition, the specific functions of the key miRNAs and functional genes still need more experimental validation.

In summary, through our study, we clarified the relationship among ncRNAs, mRNAs, and growth traits in *S. superba* and, more importantly, constructed regulatory networks that can better help researchers understand the molecular mechanisms behind the biological phenomenon of plant growth.

## 4. Materials and Methods

### 4.1. Plant Materials and RNA Sampling

*Schima superba* individuals were planted in Zijin County, Guangdong Province, in southern China (22°23′10″ E, 114°39′30″ N). The volume of wood (VW) of each individual was calculated using the tree height (HT) and diameter at breast height (DBH) with the following formula: VW = 6.01228 × 10^−5^ × DBH^1.8755^ × HT^0.98496^, presented in Table S7. After obtaining these measurements, 10 individuals with different VWs were selected for plant material collection. Mature leaves and branches of the 10 individuals were collected separately on 26 May 2021. The collected samples were immediately frozen in liquid nitrogen and stored at −80 °C before use.

Isolation of the total RNA from each sample was conducted using an RNAprep Pure Plant Kit (Tiangen, Beijing, China). The RNA samples were first qualified using 1% agarose gel electrophoresis. RNA purity and concentration were then examined using a NanoPhotometer^®^ spectrophotometer (IMPLEN, Westlake Village, CA, USA). RNA integrity and quantity were measured using an RNA Nano 6000 Assay Kit for the Bioanalyzer 2100 system (Agilent Technologies, Santa Clara, CA, USA). A total of 20 RNA samples were obtained in this step. Then, the RNA samples of leaves and branches from the same individuals were mixed equally to obtain an RNA mixture that represented the total RNA of the individual, which was used for whole-transcriptome sequencing.

### 4.2. Library Preparation and Sequencing

RNA libraries for lncRNA-seq, circRNA-seq, and mRNA-seq were prepared using rRNA depletion and the stranded method [52]. The ribosomal RNA was depleted from 5 μg of the total RNA of each sample using an rRNA removal kit following the manufacturer’s instructions. The sequencing libraries were generated using a NEBNext^®^ Ultra™ Directional RNA Library Prep Kit for Illumina^®^ (NEB, Ipswich, MA, USA) following the manufacturer’s instructions. A total of 2 μg of total RNA was used for the miRNA library preparation of each sample using a NEBNext^®^ Multiplex Small RNA Library Prep Set for Illumina^®^ (NEB, Ipswich, MA, USA) following the manufacturer’s instructions. After library preparation, the samples were sequenced on an Illumina^®^ NovaSeq 6000 platform (Illumina, San Diego, CA, USA). For lncRNAs, mRNAs, and circRNAs, 150 bp paired-end reads were generated. For miRNAs, 50 bp single-end reads were generated.

### 4.3. Quality Control

Raw data were first processed through in-house Perl scripts. For the four types of RNAs, clean data were obtained by removing reads with 5′ adapter contaminants, reads without 3′ adapter or insert sequence, reads with more than 10% N, reads with more than 50% nucleotides with Q ≤ 20%, and reads with ploy-N (A/T/C/G). Then, the Q20, Q30, and GC contents of the clean data were calculated, and the clean data were prepared for downstream analyses.

### 4.4. Read Mapping and Assembly

For lncRNAs and mRNAs, paired-end clean reads for each sample were aligned to the *S. superba* reference genome with HISAT2 software (v2.0.5) [2,53]. Read alignment results were transferred to the StringTie program for transcript assembly [54], and HISAT2 was used to identify the transcripts. For circRNAs, the index of the reference genome was built using Bowtie2 v2.2.8, and paired-end clean reads were aligned to the reference genome using Bowtie [55]. For miRNAs, reads with a length of 18–30 nt were mapped to the reference genome and analyzed using the Bowtie package [56].

### 4.5. Identification of mRNAs, lncRNAs, circRNAs, and miRNAs

All transcripts were merged using Cuffmerge software v1.0.0 [57]. mRNAs were identified using annotation information from the *S. superba* genome and transcriptome data. lncRNAs were identified from the assembled transcripts after removal of the transcripts with protein-coding capabilities using the Coding-Non-Coding Index (CNCI), Pfam, and Coding Potential Calculator (CPC) databases [58,59,60]. Then, lncRNAs were checked using the following steps: (1) removal of lowly expressed transcripts with fragments per kilobase of transcript sequence per million base pairs (FPKM) < 0.5; (2) removal of short transcripts < 200 bp and <2 exons; and (3) removal of transcripts mapped within 1-kb flanking regions of an annotated gene using Cuffcompare [57]. The targets of lncRNAs were predicted using cis-acting and trans-acting target gene prediction. The circRNAs were detected and identified using find_circ and CIRI2 [61,62]. The targets of circRNA were identified using psRobot [63]. MiRdeep2 was used to identify conserved miRNAs by comparison to miRBase and srna-tools-cli was used to draw the secondary structures of miRNA precursors [64,65,66]. The full names of the species used in miRNA naming are listed in Table S8. miREvo and MiRdeep2 were used to predict novel miRNAs using information on the hairpin structure [64,67]. The software Targetfinder v1.6 was used to predict the target genes of miRNAs [68].

### 4.6. Quantification and Differential Expression Analysis

StringTie v1.3.3 and featureCounts v1.5.0 were used to count the lncRNA and mRNA read numbers mapped to each gene with FPKM [54,69]. Cuffdiff or edgeR was used for the differential expression analysis of lncRNAs and mRNAs [70,71]. Benjamini and Hochberg’s method was used to control the false discovery rate [72]. The *p*-value was adjusted using the q-value, and genes with log_2_ |(fold change)| > 1 and *p*-value < 0.05 were defined as differentially expressed. The raw counts of circRNAs and miRNAs were normalized using transcripts per million (TPM). The FPKMs of lncRNAs and mRNAs and TPMs of circRNAs and miRNAs are provided in Tables S9‒S12. Differential expression analysis of circRNAs and miRNAs was performed using DESeq [73]. The *p*-value was adjusted using the q-value, and genes with log_2_ |(fold change)| > 1 and *p*-value < 0.05 were defined as differentially expressed [72]. Differential analysis was conducted between any two samples. In addition, the individuals with the highest (SS1) and lowest (SS10) volumes of wood were selected as reference samples to conduct comparative analysis with other samples and to identify differentially expressed mRNAs (DEmRNAs), lncRNAs (DElncRNAs), circRNAs (DEcircRNAs), and miRNAs (DEmiRNAs), respectively. Gene Ontology (GO) and Kyoto Encyclopedia of Genes and Genomes (KEGG) analyses of DEmRNAs, DElncRNAs, DEmiRNAs, and DEcircRNAs were carried out using the Goseq R package and KOBAS [74,75].

### 4.7. Construction of the Regulatory Network

After differential expression analyses using SS1 and SS10 as reference samples, nine pairs were identified for four types of RNAs in two reference samples, respectively. Then, the same DEmRNAs, DElncRNAs, DEmiRNAs, and DEcircRNAs that occurred in at least six pairs were regarded as targets to construct regulatory networks based on predicted pairs between lncRNAs or circRNAs and miRNAs and pairs between miRNAs and mRNAs. Cytoscape v3.7.2 software was used to visualize the regulatory networks [76].

### 4.8. Quantitative Real-Time PCR

The expression levels of candidate genes identified from regulatory networks were validated using quantitative real-time PCR (qRT-PCR). RNA samples collected from the leaves and branches of the 10 individuals were used for qRT-PCR validation. Primers for each candidate gene were designed using Primer 3 (version 4.0.0) [77]. *CYS*, *EF1-α*, *NADH*, *eIF*, and *ACTIN* were selected as internal reference genes to normalize the relative expression profiles among different samples (Figure S18) [78]. Primer sequences are given in Table S13, and primer-specific annealing temperatures ranged from 58 to 60 °C. The qRT-PCR in vitro reaction mixture (25 μL) was as follows: forward primer (0.5 μL), reverse primer (0.5 μL), cDNA (1 μL), qPCR mixture (10 μL), and ddH_2_O (13 μL). The PCR cycling conditions were as follows: 95 °C for 10 s, 58−60 °C for 30 s, and 72 °C for 20 s. Three replicate PCRs were performed for each gene across 10 samples. The relative expression levels of each candidate gene were calculated using the 2^−ΔΔCt^ method [79].

### 4.9. Phylogenetic Analysis

The protein of each gene was obtained from the *S. superba* genome library. Then, phylogenetic analysis of all proteins was conducted using the maximum likelihood method in Mega X with 1000 replicates [80]. The phylogenetic tree was displayed using iTOL [81].

### 4.10. SNP Analysis

GATK v3.5 software was used to perform single nucleotide polymorphism (SNP) calling, and SnpEff software v4.5 was used for variable site annotation [82,83].

### 4.11. Heatmap and Gene Analysis

The heatmap of gene expression across the 10 samples was drawn using FPKM values in RNA-seq. A correlation analysis was conducted and drawn using GraphPad Prism 8.3.0. MEME was used to identify conserved motifs in the coding sequences [84]. Gene structures, motif exhibitions, and circos plots with annotations were drawn using TBtools [85].

## 5. Conclusions

Growth is one of the most important traits in *S. superba* breeding and practical production. In this study, whole-transcriptome technology was employed to identify the key regulators and genes related to growth traits. A total of 32,711 mRNAs, 525 miRNAs, 54,312 lncRNAs, and 1522 circRNAs were identified from 10 individuals with different VWs. The construction of regulatory networks indicated that the lncRNAs TCONS_00049201, TCONS_00121864, TCONS_00141936, and circRNA novel_circ_0000702 are possible regulators of growth traits. Eleven miRNAs, aly-miR157d-3p, aly-miR172e-3p, ata-miR395b-3p, bdi-miR159a-3p, gma-miR396a-3p, gma-miR6300, hbr-miR156, novel_102, osa-miR396a-3p, sbi-miR172b, and gma-miR408d, can be considered core mediators in the modules of lncRNA–miRNA–mRNA and circRNA–miRNA–mRNA networks. Several transcription factors, including *SsDELLA2* (*SsSLR*), *SsDELLA3* (*SsSLN*), *SsDELLA5* (*SsGAI-like2*), and *SsNAM1*, and functional genes, including *Ss4CL2*, *SsCSL1*, and *SsCSL2*, are likely directly involved in the biosynthesis of lignin and cellulose. In addition, 86,922 SNPs were identified from the transcript sequences across the 10 samples. Our study provides new insights into the molecular mechanism underlying the growth traits of *S. superba* and massive allelic variations that will benefit molecular breeding work. These candidate genes may be used for directed gene editing, while the allelic variations can be used for association studies with growth traits and marker-assisted selection.

## Figures and Tables

**Table 1:** Percentage of transition and transversion single nucleotide polymorphisms (SNPs) using RNA-seq data.

SNP Type	Transition SNPs		Transversion SNPs			
	C/T	A/G	A/T	A/C	G/T	G/C
Number of allelic sites	26,704	26,579	9163	8453	8444	7579
Frequency (%)	30.72	30.58	10.54	9.73	9.71	8.72
Total (percent of total)	53,283 (61.30%)		33,639 (38.70%)			

## Data Availability

All the data have been deposited in the NCBI Sequence Read Archive (SRA) database with accession number of PRJNA1007219. The script used in the manuscript will be made available upon request.

## Supplementary Materials

The following supporting information can be downloaded at https://www.mdpi.com/article/10.3390/ijms25042171/s1.
