# Supplementary material for: The Construction of lncRNA/circRNA–miRNA–mRNA Networks Reveals Functional Genes Related to Growth Traits in Schima superba
Source: Int J Mol Sci. 2024 Feb 11;25(4):2171. doi: 10.3390/ijms25042171 (PMC10888550; doi:10.3390/ijms25042171)
Supplement: Supplementary file 1 [file ijms-25-02171-s001.zip › Supplementary Figures.pdf]

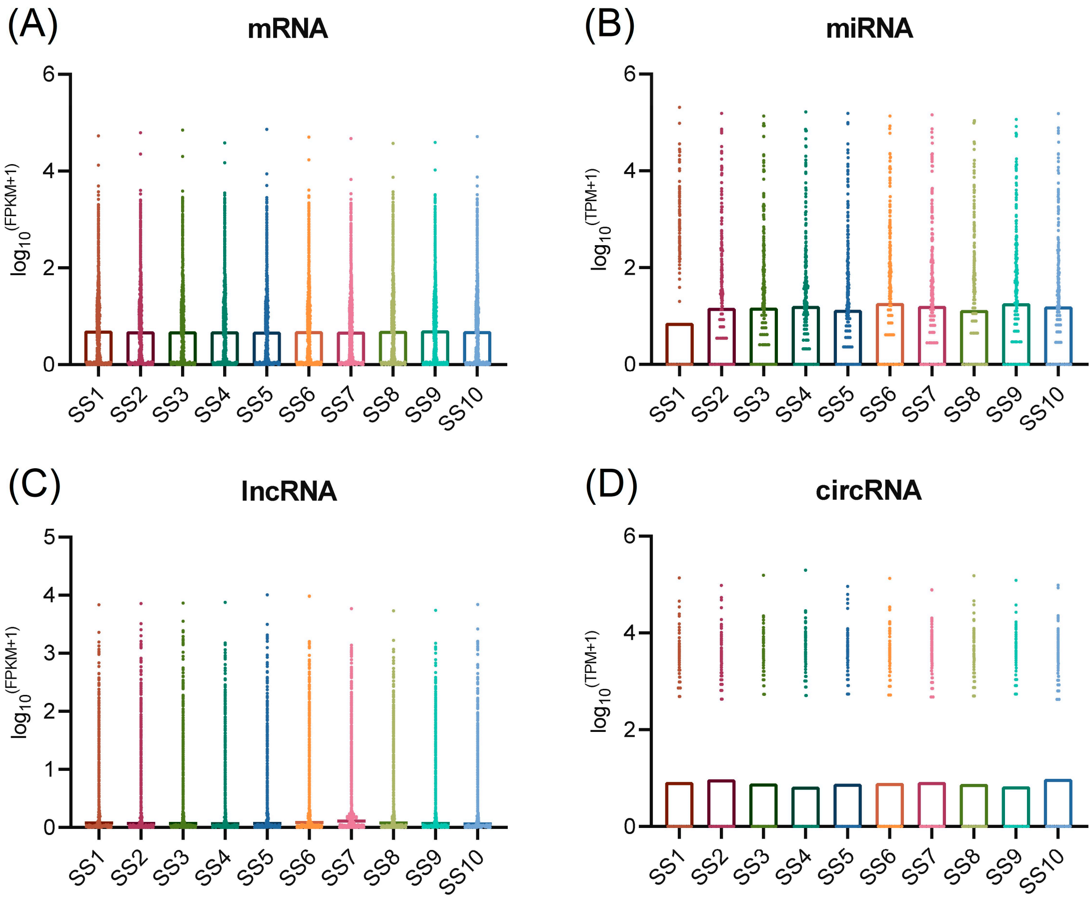

Figure S1 Expression profiles of four types of RNAs using whole transcriptome sequencing. (A) Distribution of mRNA Fragments Per Kilobase of exon model per Million mapped fragments (FPKM) values across 10 *Schima superba* individuals with different volume of wood (VW). (B) Distribution of miRNA Transcripts Per Kilobase Million (TPM) values across 10 *S. superba* individuals with different VWs. (C) Distribution of lncRNA FPKM values across 10 *S. superba* individuals with different VWs. (D) Distribution of circRNA TPM values across 10 *S. superba* individuals with different VWs. The original data of FPKM and TPM values of four types of RNAs was transformed using the following formula: $\log_{10}^{(FPKM/TPM+1)}$. Top of the boxes represent mean values of each sample.

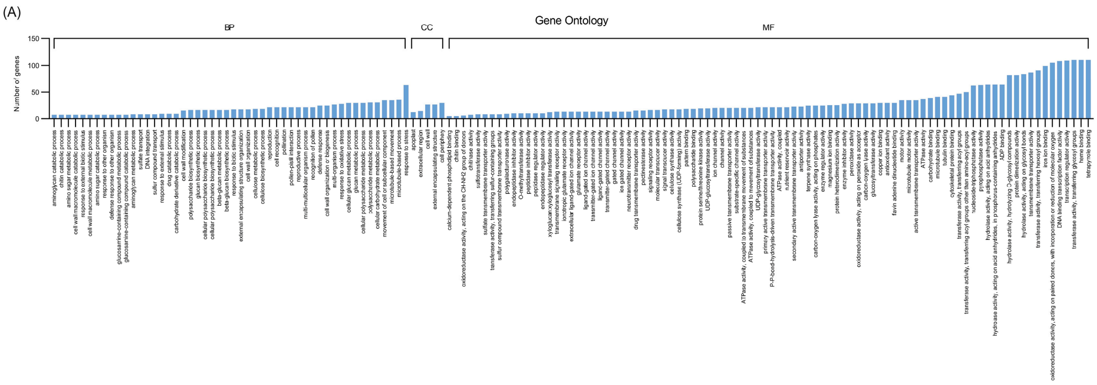

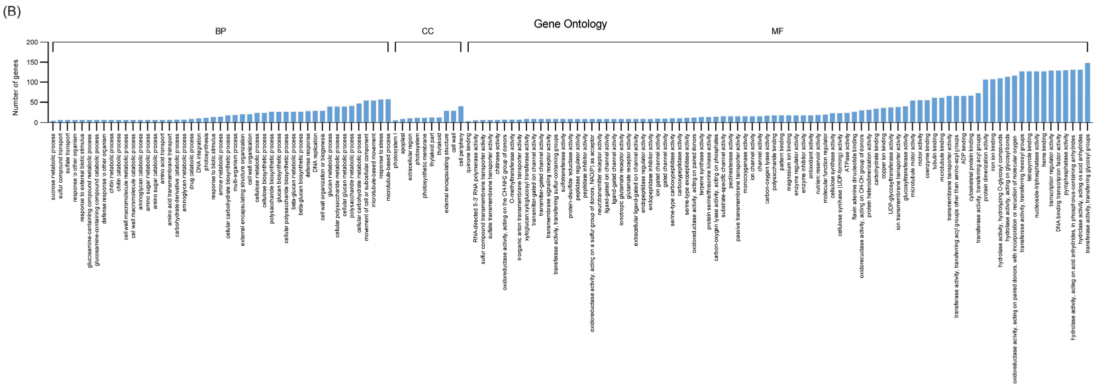

Figure S2 Gene Ontology (GO) classification of differentially expressed mRNAs (DEmRNAs) among 10 individuals. (A) GO terms of all the DEmRNAs obtained from nine comparative pairs using SS1 as reference sample. (B) GO terms of all the DEmRNAs obtained from nine comparative pairs using SS10 as reference sample. In each comparative pair using SS1 or SS10 as reference, GO classification with the largest number of DEmRNAs was listed in both (A) and (B).

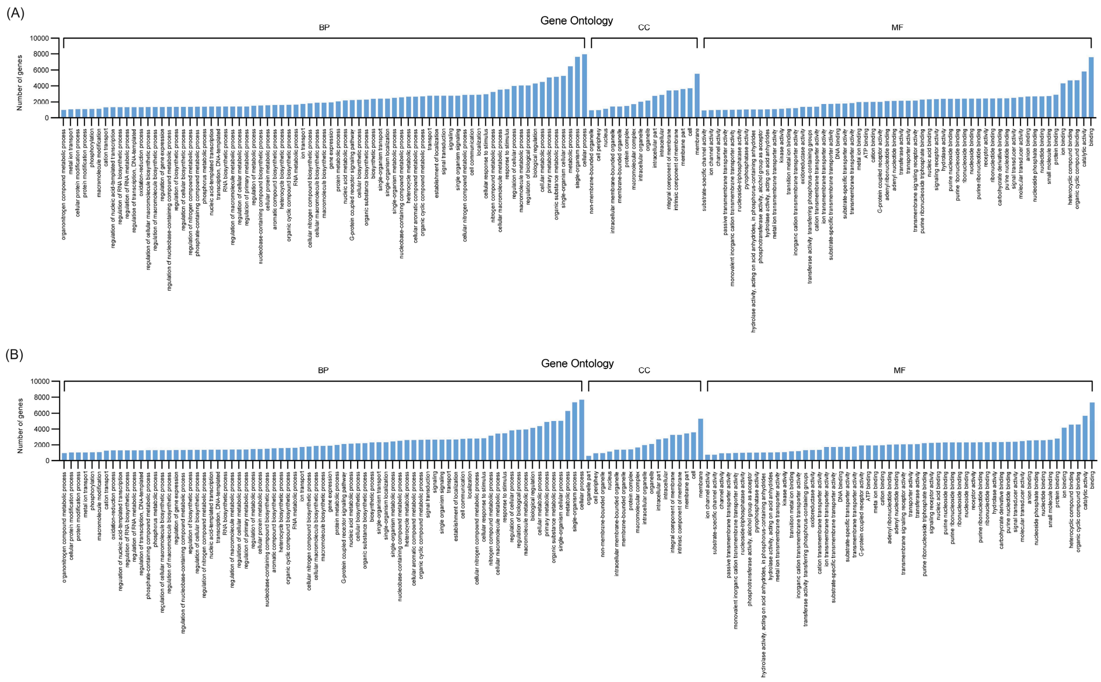

Figure S3 Gene Ontology (GO) classification of differentially expressed miRNAs (DEmiRNAs) among 10 individuals. (A) GO terms of all the DEmiRNAs obtained from nine comparative pairs using SS1 as reference sample. (B) GO terms of all the DEmiRNAs obtained from nine comparative pairs using SS10 as reference sample. In each comparative pair using SS1 or SS10 as reference, GO classification with the largest number of DEmRNAs was listed in both (A) and (B).

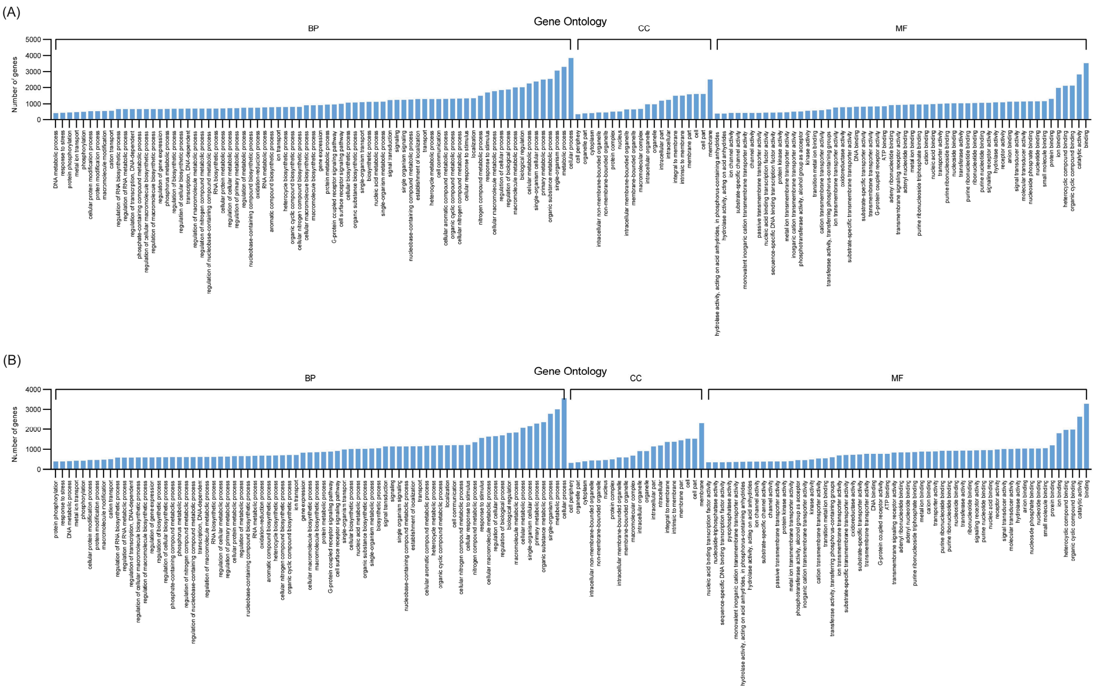

Figure S4 Gene Ontology (GO) classification of differentially expressed lncRNAs (DElncRNAs) among 10 individuals. (A) GO terms of all the DElncRNAs obtained from nine comparative pairs using SS1 as reference sample. (B) GO terms of all the DElncRNAs obtained from nine comparative pairs using SS10 as reference sample. In each comparative pair using SS1 or SS10 as reference, GO classification with the largest number of DElncRNAs was listed in both (A) and (B).

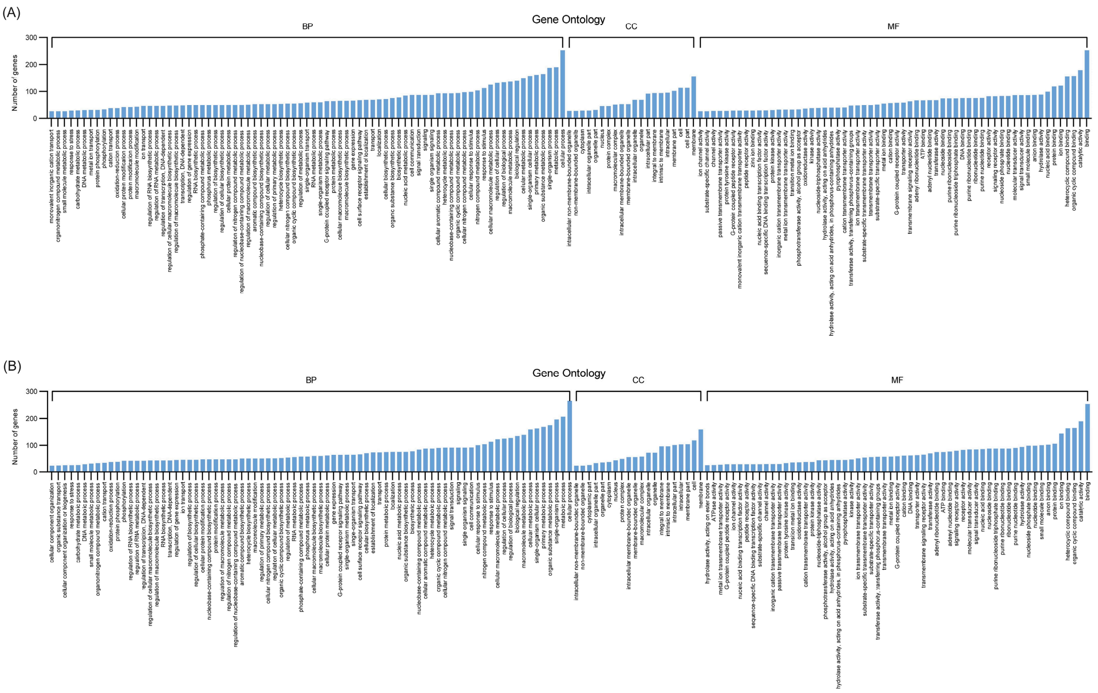

Figure S5 Gene Ontology (GO) classification of differentially expressed circRNAs (DEcircRNAs) among 10 individuals. (A) GO terms of all the DEcircRNAs obtained from nine comparative pairs using SS1 as reference sample. (B) GO terms of all the DEcircRNAs obtained from nine comparative pairs using SS10 as reference sample. In each comparative pair using SS1 or SS10 as reference, GO classification with the largest number of DEcircRNAs was listed in both (A) and (B).

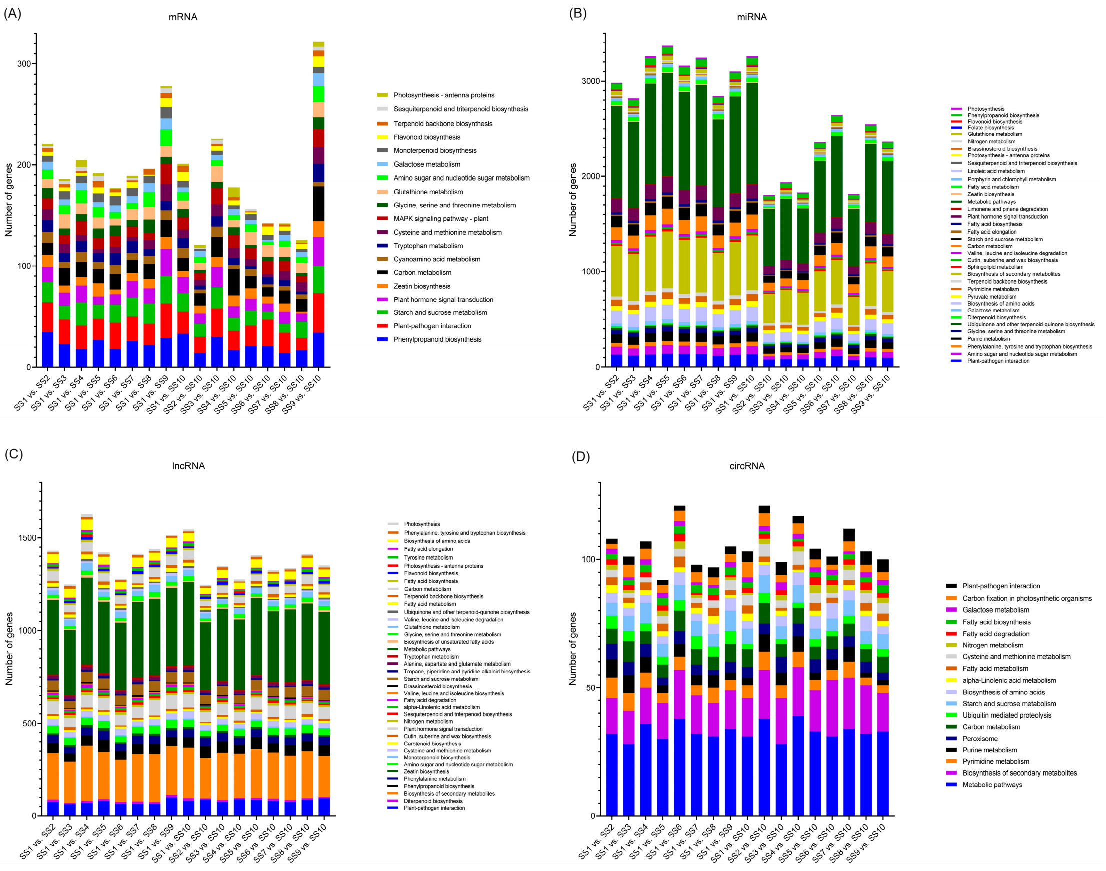

Figure S6 Kyoto Encyclopedia of Genes and Genomes (KEGG) classification of differentially expressed mRNAs (DEmRNAs), miRNAs (DEmiRNAs), lncRNAs (DElncRNAs), and circRNAs (DEcircRNAs) among 10 individuals. (A) Stacked column chart of DEmRNA KEGG classifications among 10 individuals using SS1 and SS10 as reference samples. (B) Stacked column chart of DEmiRNA KEGG classifications among 10 individuals using SS1 and SS10 as reference samples. (C) Stacked column chart of DElncRNA KEGG classifications among 10 individuals using SS1 and SS10 as reference samples. (D) Stacked column chart of DEcircRNA KEGG classifications among 10 individuals using SS1 and SS10 as reference samples.

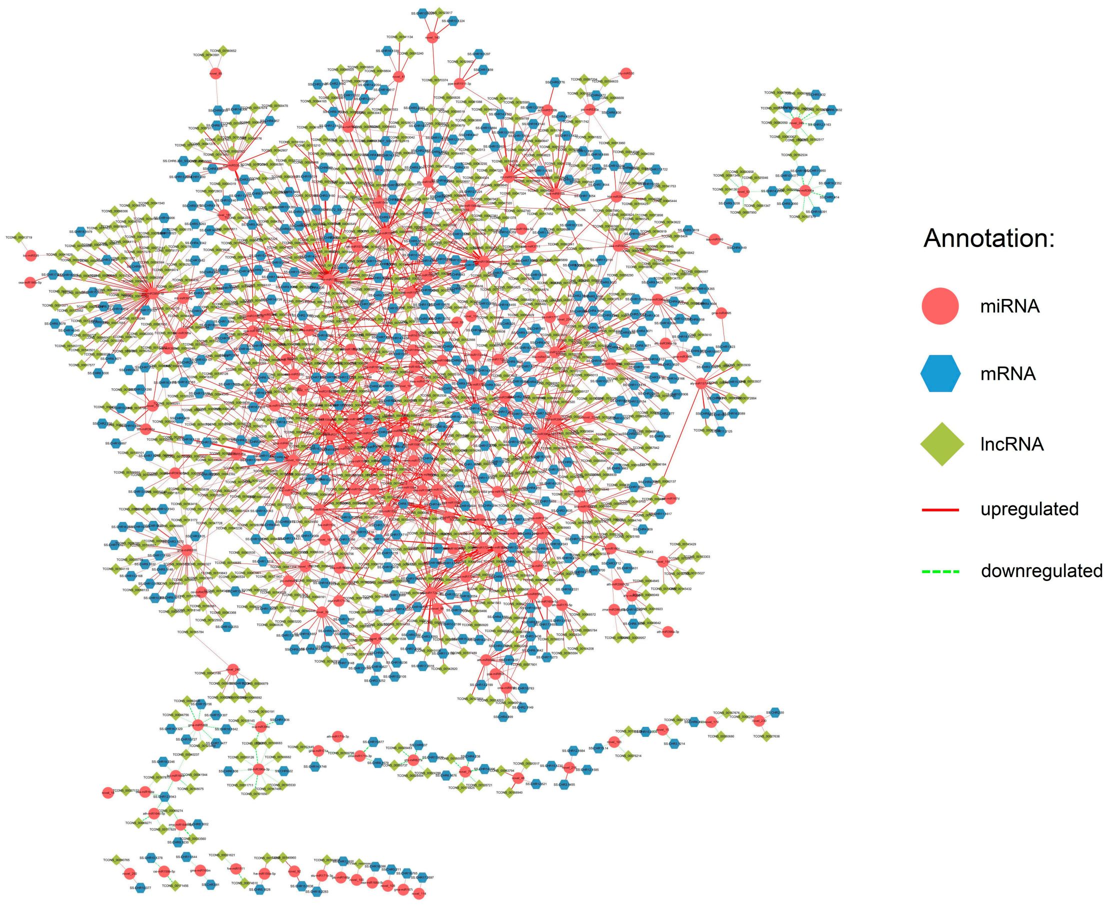

Figure S7 Regulatory network of lncRNA-miRNA-mRNA compared to SS1. Differential analysis was conducted using the individual with highest volume of wood (SS1) as control. Across nine comparison pairs, three types of differentially expressed RNAs that simultaneously detected in over six pairs were used for construction of regulatory network. Solid red lines meant upregulated RNAs, while dotted green lines meant downregulated RNAs. The thicker the line, the higher number of comparison pairs was used.

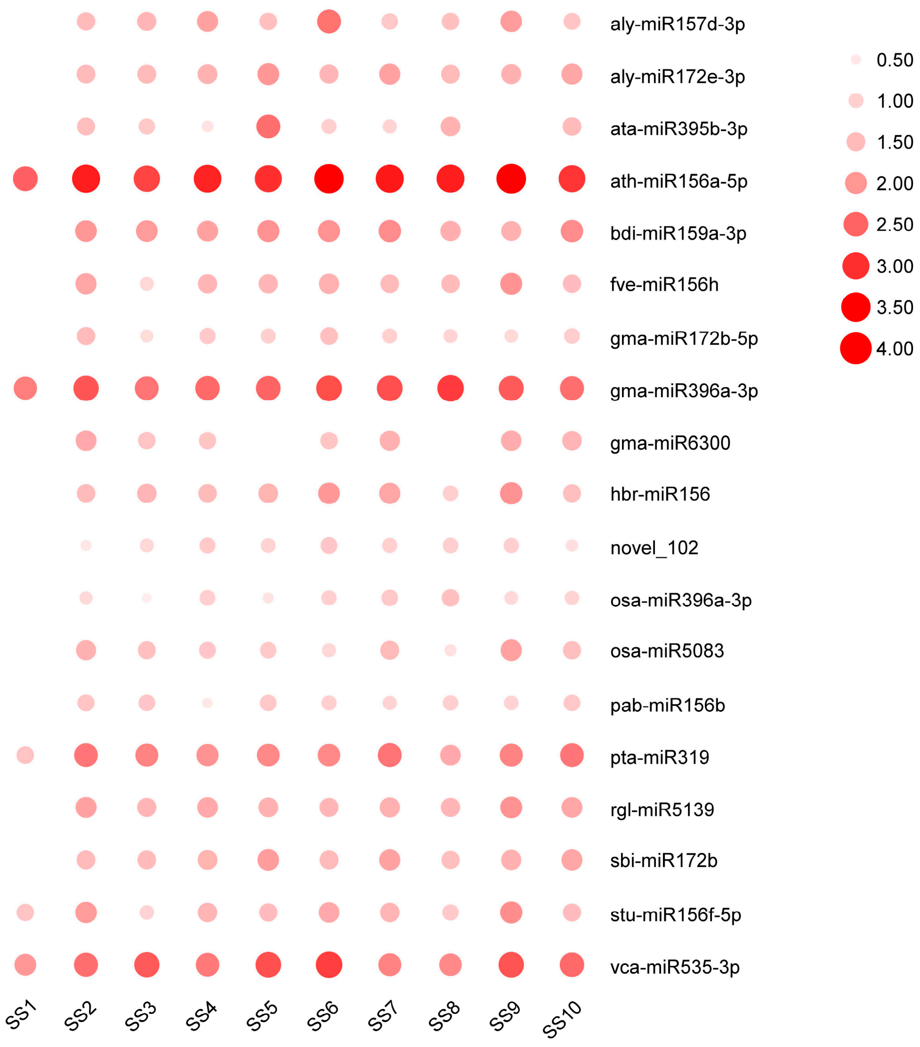

Figure S8 Heatmap of 19 miRNAs in the network of lncRNA-miRNA-mRNA in comparison to SS1. TPM values of each miRNA in ten individuals were transformed using $\log_{10}^{TPM}$ and displayed by TBtools. The larger the circle size and the darker the color, the higher the expression levels.

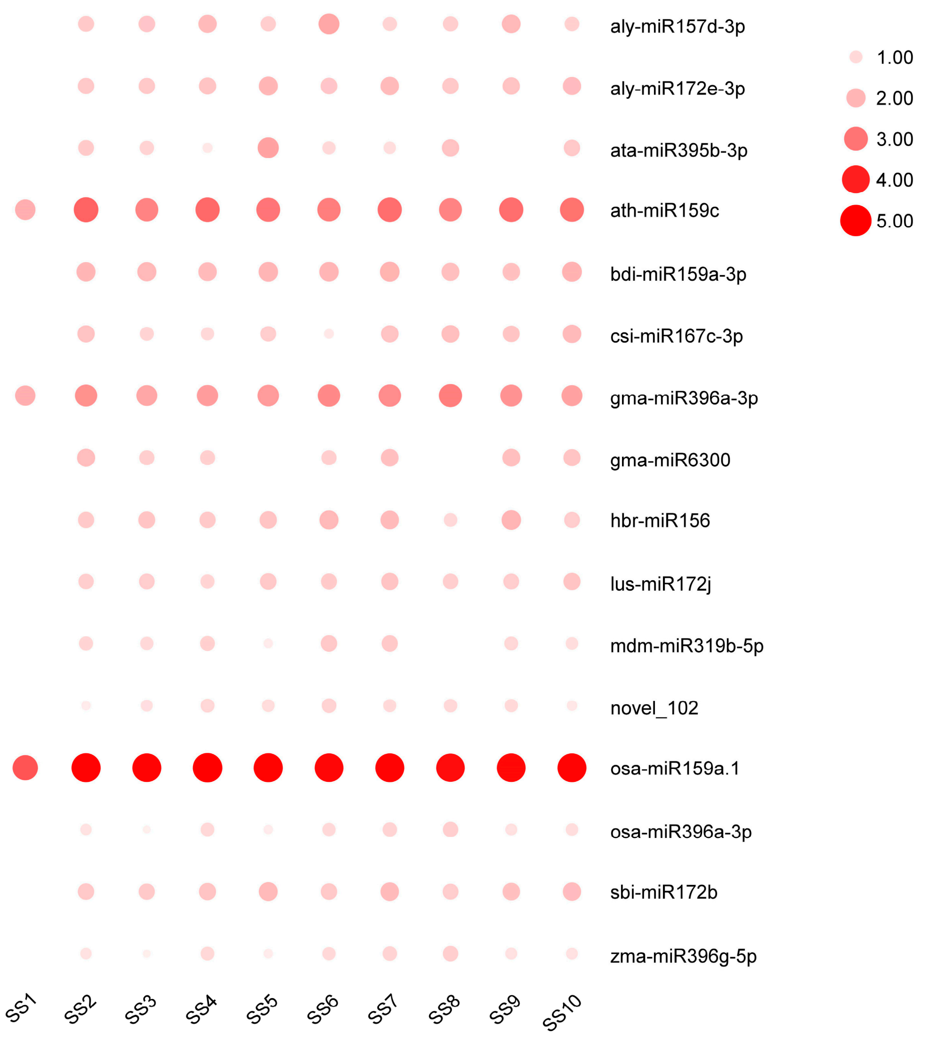

Figure S9 Heatmap of 16 miRNAs in the network of circRNA-miRNA-mRNA in comparison to SS1. TPM values of each miRNA in ten individuals were transformed using log_{10}^{TPM} and displayed by TBtools. The larger the circle size and the darker the color, the higher the expression levels.

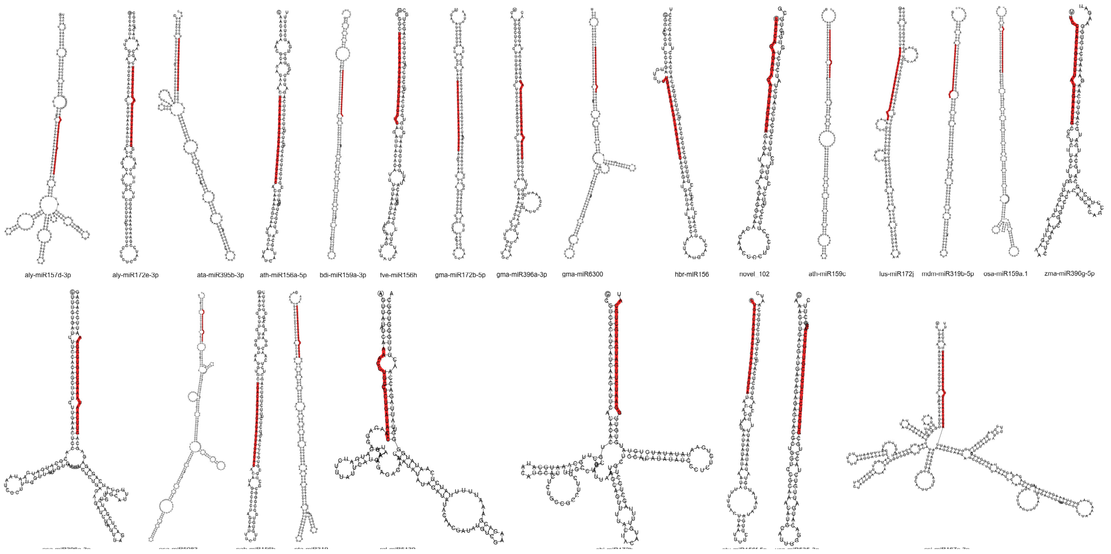

Figure S10 Secondary structure of 25 key miRNAs in the networks of lncRNA-miRNA-mRNA and circRNA-miRNA-mRNA. The whole sequence represents the precursor of miRNA, and the sequence highlighted with red color represents mature sequence.

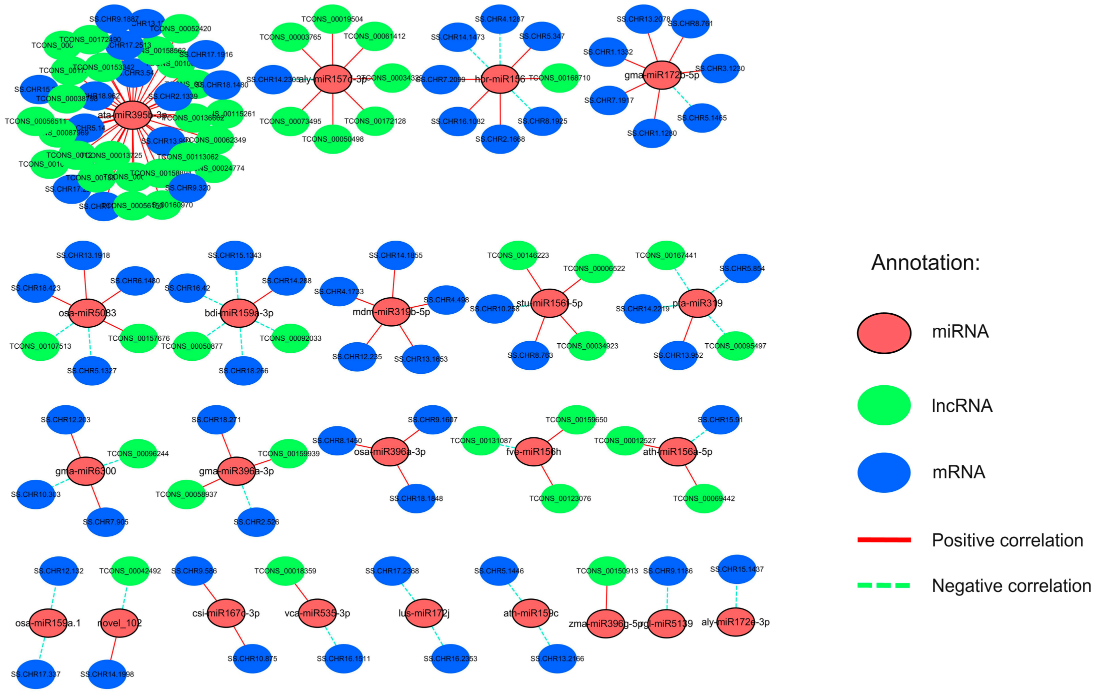

Figure S11 Regulatory network of lncRNA-miRNA-mRNA using correlation analysis. The solid red connection lines represent positive correlations and dotted green lines represent negative correlations. Connections with correlation coefficients of positive correlations ≥ 0.8 or negative correlations ≤ −0.8 were displayed ($p < 0.01$).

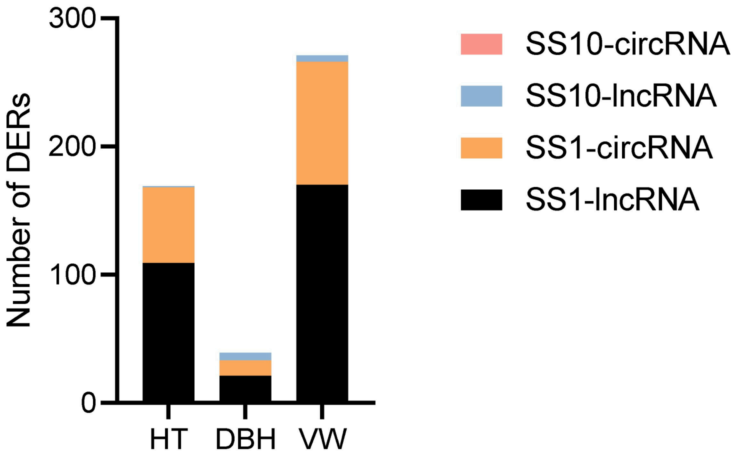

Figure S12 Number of correlated RNAs (lncRNAs and circRNAs) with growth trait in the regulation networks of using SS1 and SS10 as reference samples. The phenotypes of HT, DBH, and VW were used for analysis and statistic, respectively.

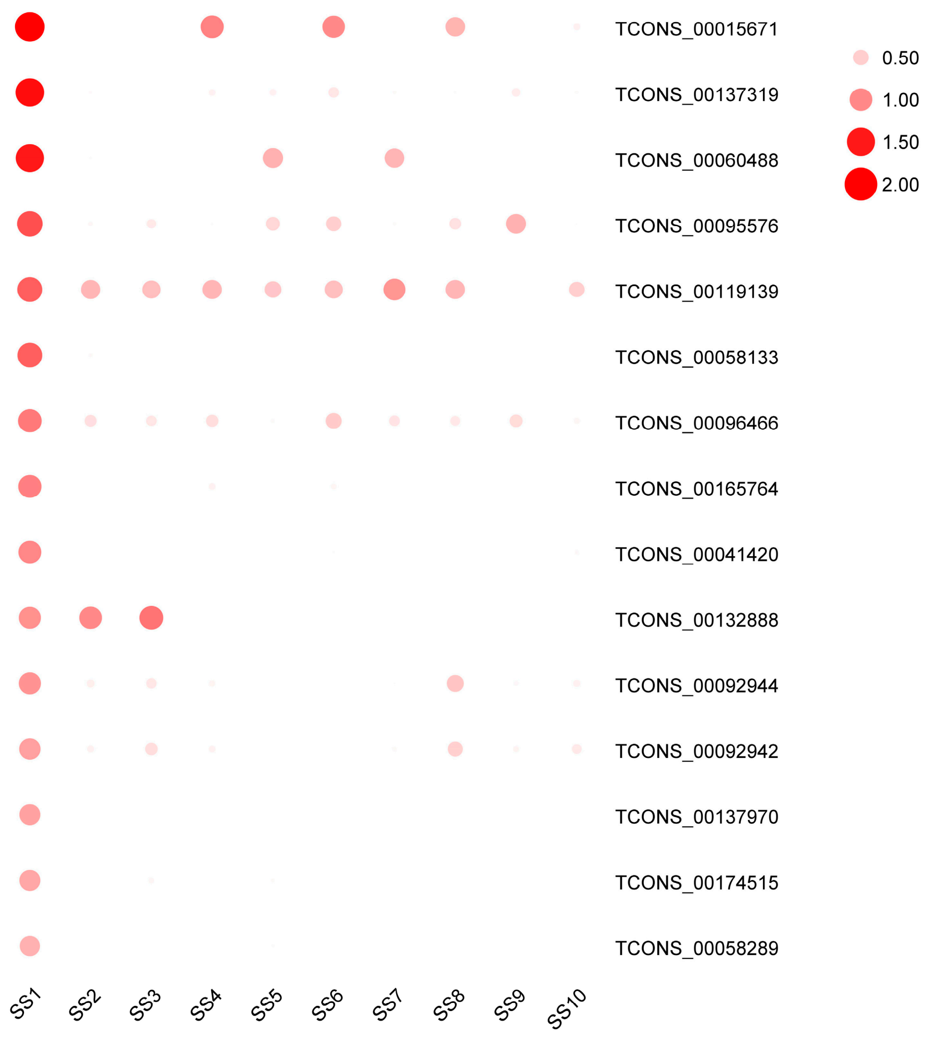

Figure S13 Heatmap of 15 lncRNAs in the network of lncRNA-miRNA-mRNA. FPKM values of each lncRNA in ten individuals were transformed using $\log_{10}^{TPM}$ and displayed by TBtools. The larger the circle size and the darker the color, the higher the expression levels.

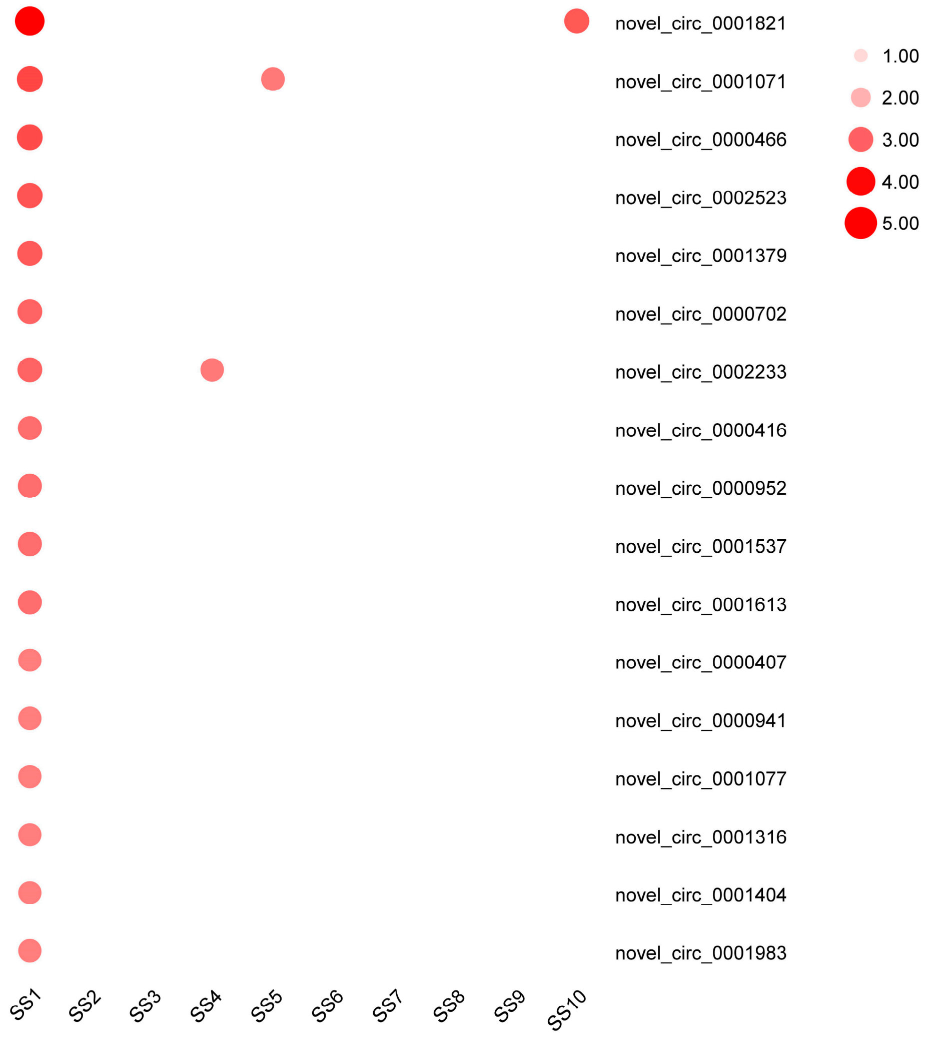

Figure S14 Heatmap of 17 miRNAs in the network of circRNA-miRNA-mRNA. TPM values of each circRNA in ten individuals were transformed using $\log_{10}^{TPM}$ and displayed by TBtools. The larger the circle size and the darker the color, the higher the expression levels.

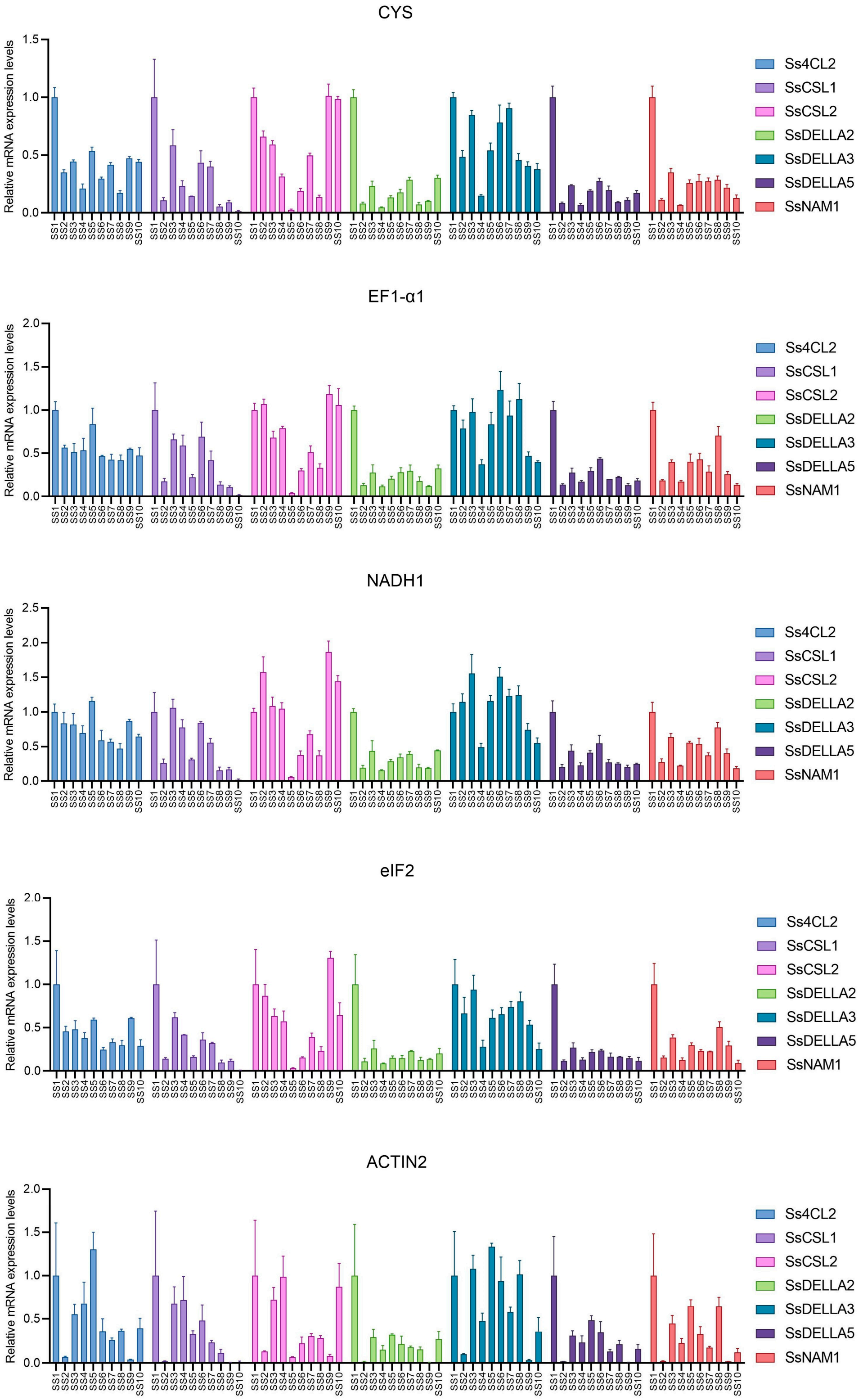

Figure S15 qRT-PCR validation of seven potential genes in branches across 10 individuals. The branches were collected from 10 individuals and used for qRT-PCR validation. A total of seven candidate genes were used for qRT-PCR with five references.

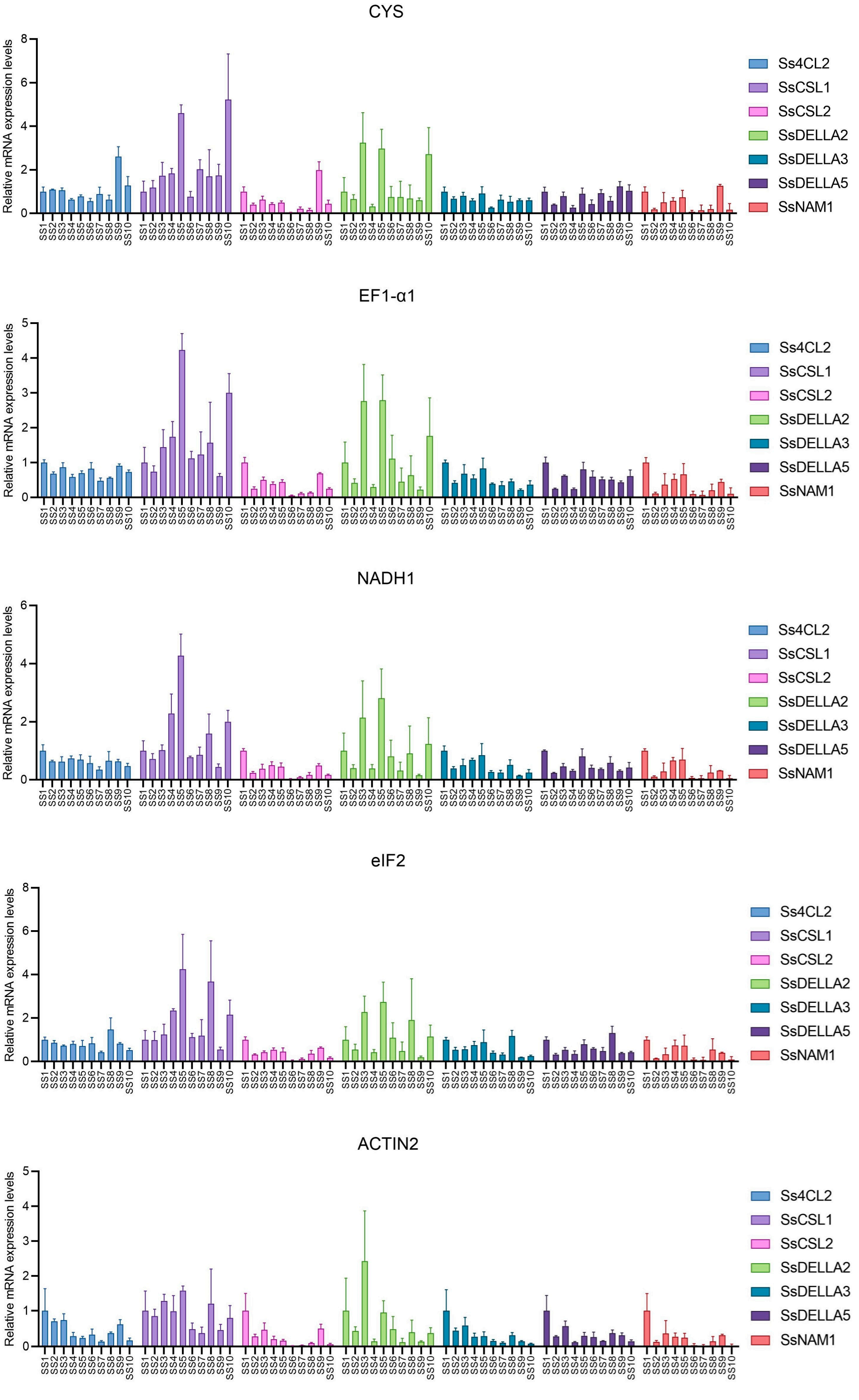

Figure S16 qRT-PCR validation of seven potential genes in leaves across 10 individuals. The leaves were collected from 10 individuals and used for qRT-PCR validation. A total of seven candidate genes were used for qRT-PCR with five references.

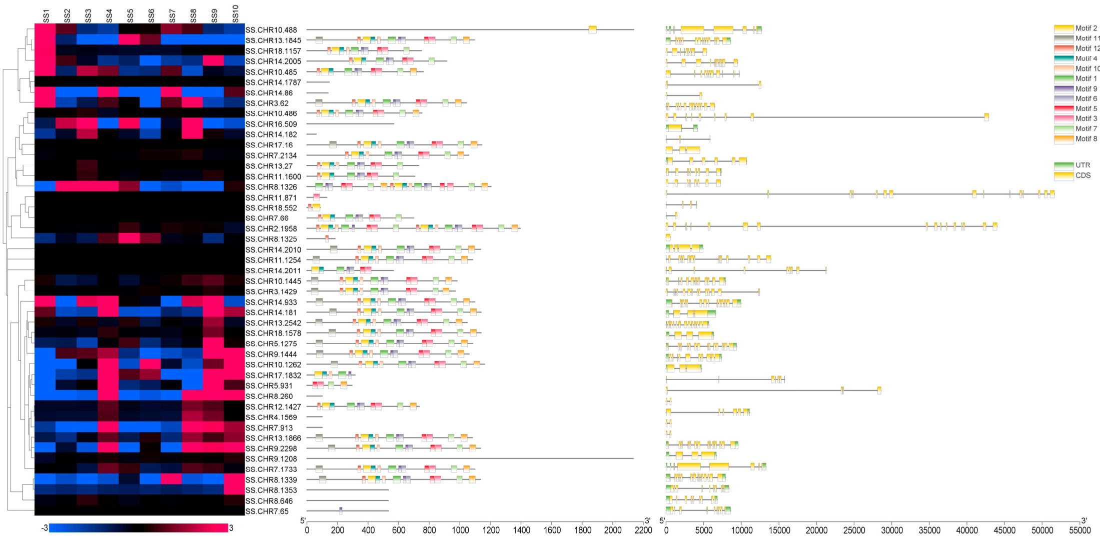

Figure S17 Transcriptomic expression, motif, and gene structure of cellulose synthase and cellulose synthase-like genes. Heapmap was used to display the gene transcriptomic expression of 47 cellulose synthase and cellulose synthase-like genes. Motifs of these genes were identified using MEME (https://meme-suite.org/meme/). Then, TBtools were used to combine and display the heatmap, motif, and gene structure of these genes.

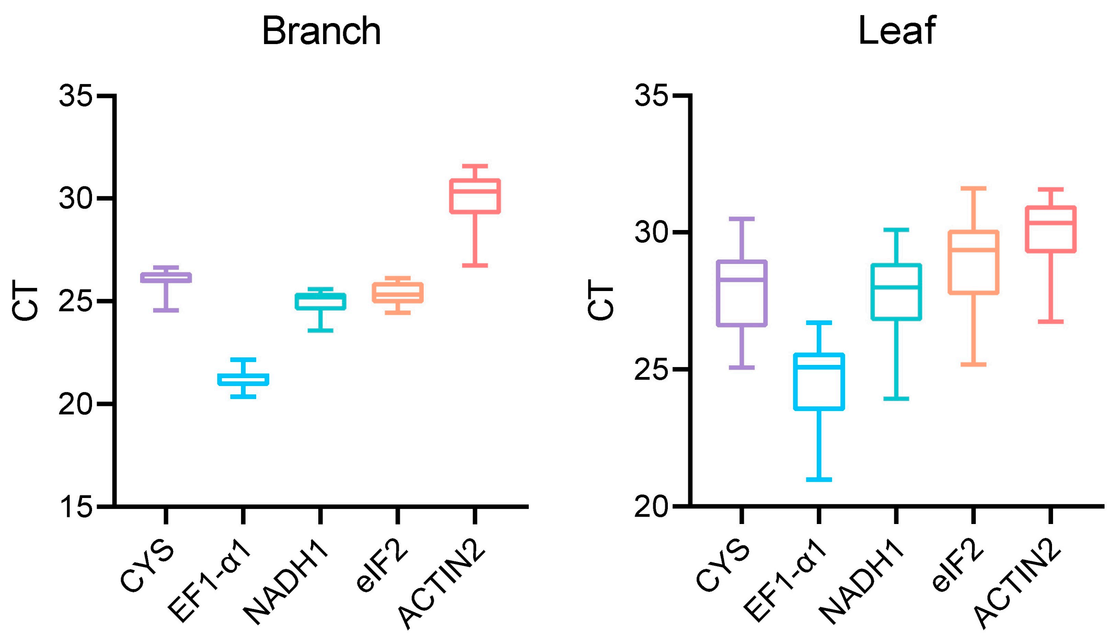

Figure S18 The box plot of CT values of five reference genes used for qRT-PCR validation. A total of 18 candidate reference genes were used to select suitable reference genes for growth trait in *Schima superba*. The candidate functional genes were all normalized by five reference genes (CYS, EF1-α1, NADH1, eIF2, and ACTIN2).
